# The effect of bile acids on the growth and global gene expression profiles in *Akkermansia muciniphila*

**DOI:** 10.1007/s00253-020-10976-3

**Published:** 2020-11-07

**Authors:** Tatsuro Hagi, Sharon Y. Geerlings, Bart Nijsse, Clara Belzer

**Affiliations:** 1 Laboratory of Microbiology, Wageningen University and Research, 6708 WE Wageningen, The Netherlands; 2 Institute of Livestock and Grassland Science, National Agriculture and Food Research Organization (NARO), 2 Ikenodai, Tsukuba, 305-0901 Ibaraki, Japan; 3 Laboratory of Systems and Synthetic Biology, Wageningen University and Research, 6708 WE Wageningen, The Netherlands

**Keywords:** *Akkermansia muciniphila*, Bile acids, Transcriptome

## Abstract

**Abstract:**

*Akkermansia muciniphila* is a prominent member of the gut microbiota and the organism gets exposed to bile acids within this niche. Several gut bacteria have bile response genes to metabolize bile acids or an ability to change their membrane structure to prevent membrane damage from bile acids. To understand the response to bile acids and how *A*. *muciniphila* can persist in the gut, we studied the effect of bile acids and individual bile salts on growth. In addition, the change in gene expression under ox-bile condition was studied. The growth of *A*. *muciniphila* was inhibited by ox-bile and the bile salts mixture. Individual bile salts have differential effects on the growth. Although most bile salts inhibited the growth of *A*. *muciniphila*, an increased growth was observed under culture conditions with sodium deoxycholate. Zaragozic acid A, which is a squalene synthase inhibitor leading to changes in the membrane structure, increased the susceptibility of *A*. *muciniphila* to bile acids. Transcriptome analysis showed that gene clusters associated with an ABC transporter and RND transporter were upregulated in the presence of ox-bile. In contrast, a gene cluster containing a potassium transporter was downregulated. Membrane transporter inhibitors also decreased the tolerance to bile acids of *A*. *muciniphila*. Our results indicated that membrane transporters and the squalene-associated membrane structure could be major bile response systems required for bile tolerance in *A. muciniphila*.

**Key points:**

• *The growth of Akkermansia muciniphila was inhibited by most bile salts.*

• *Sodium deoxycholate increased the growth of A. muciniphila.*

• *The genes encoding transporters and hopanoid synthesis were upregulated by ox-bile.*

• *The inhibitors of transporters and hopanoid synthesis reduced ox-bile tolerance.*

**Supplementary Information:**

The online version contains supplementary material available at 10.1007/s00253-020-10976-3.

## Introduction

The human gut microbiome study has revealed the biodiversity of gut bacteria in healthy individuals or individuals with human (Consortium HMP 2012). A part of the gut bacteria plays an important role in human health (Flint et al. 2012) and interacts with host immunity and nutrition (Rowland et al. 2018; Thaiss et al. 2016). *Akkermansia muciniphila* is an attractive commensal gut bacteria to study because of its probiotic effect such as improvement of obesity and metabolic disorders and modulation of host immunity (Ansaldo et al. 2019; Everard et al. 2013). *A*. *muciniphila* resides in the colonic mucus layer where this bacterium degrades mucin, using it as carbon and nitrogen source (Derrien et al. 2004). The mucus layer, which is mainly composed of glycoproteins with specific *O*-linked glycans, is a major host defense system. Other commensal and pathogenic bacteria have adhesive molecules to mucus for gut colonization (Sicard et al. 2017). Some factors such as intestinal pH, oxygen, and bile acids also influence microbial composition in the colon (Flint et al. 2012). Oxygen generated by epithelial cells could cause damage to strict anaerobic gut bacteria. In addition to a nutritional advantage based on mucin utilization, *A*. *muciniphila* also has an oxygen consumption system using the cytochrome bd complex (Ouwerkerk et al. 2016). *A*. *muciniphila* takes an advantage of this unique system to protect the cell from oxygen damage and thereby persists in the gut. Recently, it is reported that *A*. *muciniphila* needs GlcNAc for growth in the absence of mucus, and a defined medium without mucin, which supports understanding physiological properties of *A*. *muciniphila*, has been established (van der Ark et al. 2018).

The interaction between bile acids and gut bacteria is extremely complex. Bile acids consist of primary bile acids produced in the liver such as cholic acid (CA) and chenodeoxycholic acid (CDCA) and secondary bile acids generated by gut bacteria such as deoxycholic acid (DCA) and lithocholic acid (LCA) (Wahlstrom et al. 2016). There is a strong relationship between bile acids and human health (Wahlstrom et al. 2016). Secondary bile acids can cause colitis (Saracut et al. 2015). On the other hand, secondary bile acids can inhibit the growth of *Clostridium difficile* causing diarrhea and colitis in mice (Studer et al. 2016). The balance of bile acids and microbiota is also important for our health because dysbiosis leading to secondary bile acid deficiency could promote intestinal inflammation (Sinha et al. 2020). To clear the complex metabolism of bile acids, the interaction of bile acid–gut bacteria axis and its impacts on human health and disease have been widely studied (Staley et al. 2017; Wahlstrom et al. 2016).

*A*. *muciniphila* is one of the most related commensal gut bacteria to bile acid–host metabolism axis. Van den Bossche et al. (2017) reported that the administration of ursodeoxycholic acid, which is a small amount in the human intestine, could increase a number of *A*. *muciniphila* and improve colitis in mice. The increase of bile acids (especially, cholic acid) could reduce the population of *A*. *muciniphila* in the high-fat diet–induced obese mice (Zheng et al. 2017), and this study indicates *A*. *muciniphila* may be regulated by bile acids. The investigation of cross talk between *A*. *muciniphila* and bile acids is important to understand how *A*. *muciniphila* can survive in the gut environment and contribute to human health.

Bile acids could damage the bacterial cell membrane and thereby cause cell death (Kurdi et al. 2006). Therefore, bile acid tolerance of probiotic bacteria has been widely investigated since bile acid tolerance leading to survivability in the gut is one of the probiotic criteria. Bile acid metabolism in *Lactobacillus* and *Bifidobacterium* has been reviewed (Ruiz et al. 2013). Changes in membrane components such as isoprenoid and peptidoglycan are related to bile acid tolerance in Gram-positive bacteria such as *Listeria* and lactic acid bacteria (Begley et al. 2002; Hagi et al. 2013; Hamon et al. 2012). In Gram-negative bacteria, hopanoids (a group of isoprenoids) are required for bile acid resistance in *Rhodopseudomonas palustris* and symbiosis with plant in *Bradyrhizobium* sp. (Hamon et al. 2012; Silipo et al. 2014; Welander et al. 2009). Isoprenoids are one of the most important factors for bile acid tolerance in both Gram-positive and Gram-negative bacteria. In addition, bile acid–inducible (*bai*) genes encoding bile transporters and dehydroxylation enzymes are also mainly related to bile acid metabolism and tolerance in the gut bacteria (Vital et al. 2019). Although a gene for a bile acid:sodium symporter (Amuc_0139) is found in the genome of *A*. *muciniphila* (NC_010655), the dynamics and gene expression of *A*. *muciniphila* in response to bile acids are unknown.

To clear how *A*. *muciniphila* interacts with bile acids, in this study, the growth of *A*. *muciniphila* under bile acids and individual bile salt condition including main bile acids such as cholic acid and deoxycholic acid was investigated. In addition, the response to bile acids was investigated by transcriptome analysis. Here, we show that the different effects of bile salts on the growth of *A*. *muciniphila* and change in gene expression have grown under bile acid condition. Furthermore, the effect of a squalene synthase inhibitor and membrane transporter inhibitors on bile acid tolerance in *A*. *muciniphila* is reported herein.

## Materials and methods

### *A*. *muciniphila* growth condition

*A*. *muciniphila* MucT (DSM 22959) was anaerobically grown in 10 mL basal medium (Derrien et al. 2004) supplemented with 20 g/L tryptone, 4 g/L L-threonine, 2.75 g/L GlcNAc, and 2.5 g/L glucose monohydrate at 37 °C (van der Ark et al. 2017). All components in modified basal medium (mBM) were purchased from Sigma-Aldrich (St. Louis, MO, USA), except for tryptone (Oxoid Ltd., Basingstoke, Hampshire, England).

Bile extracts and individual bile salts were purchased from Sigma-Aldrich: bile extract porcine (bile porcine: B8631), ox-bile for microbiology (ox-bile; 70,168), bile salts for microbiology (bile salts mixture: mixture of sodium cholate and sodium deoxycholate; B8756), sodium cholate (CA; 27028), glycocholic acid sodium (GCA; G7132), sodium deoxycholate (DCA; D6750), sodium glycochenodeoxycholate (GCDCA; G0759), sodium glycodeoxycholate (GDCA; G9910), sodium taurocholate hydrate (TCA; 86,339), sodium taurodeoxycholate hydrate (TDCA; T0557), chenodeoxycholic acid sodium (CDCA; C8261), and taurochenodeoxycholic acid sodium (TCDCA; T6260). For stock solutions, ox-bile and bile salts mixture were dissolved in mBM, and bile porcine was dissolved in distilled water at a concentration of 10% (wt/vol). Stock solutions of each individual bile salts were prepared in a medium of 100 mM (wt/vol). Stock solutions except bile porcine were sterilized by 0.22-μm-pore polyethersulfone membrane filter (mdi Membrane Technologies; Harrisburgh, PA, USA) before use. The bile porcine was adjusted to pH 7.0 by NaOH and autoclaved. Stock solutions were added to culture media in different concentrations.

One milliliter of a fully grown pre-culture containing *A*. *muciniphila* in mBM was inoculated into 10 mL of mBM supplemented with different concentrations of bile extract M and individual bile salts. To test for the inhibition of isoprenoid (squalene) production, zaragozic acid A (ZA; Santa Cruz Biotechnology, Santa Cruz, CA, USA) was added to the culture containing 0.1% ox-bile, bile salts mixture, and bile porcine. The final concentration of ZA in these cultures was 15 μM. After incubation at 37 °C for 48 h, the optical density (OD_600_) was measured as reported previously (Ouwerkerk et al. 2016). For the ZA-treated test, mBM with 70% ethanol (25.9 μL/10 mL mBM) was used as control because ZA was diluted in 70% ethanol. These experiments were performed in triplicate (*n* = 3). The statistical analysis was performed by Dunnett’s test or paired *t* test. For quality control, the cultures were visualized under the microscope after growth following 48 h of incubation.

### RNA extraction

Two milliliters of a fully grown pre-culture containing *A*. *muciniphila* in mBM was inoculated into 10 mL mBM supplemented with 0.1% ox-bile. Cell cultures were grown in triplicate under control and ox-bile conditions. After incubation at 37 °C, 7 mL of cell culture (OD_600_ = ~ 1.0) was mixed with 14 mL of RNAprotect Bacteria Reagent (Qiagen GmbH, Hilden, Germany). After centrifugation at 8000×*g* for 10 min, the cell pellets were dissolved in 200 μL of TE buffer containing lysozyme (15 mg/mL), proteinase K (0.1 mg/mL), and mutanolysin (10 U/mL). After incubation for 40 min at room temperature, RTL buffer was added and the RNA extraction with DNase treatment was performed using a RNeasy mini kit and RNase-Free DNase Set according to the manufacturer’s instructions. RNA and DNA concentrations were measured using the Qubit RNA BR assay kit and the Qubit DNA BR assay kit, respectively (Thermo Fisher Scientific, Waltham, Massachusetts, USA). The quality of the isolated RNA was assessed using a Qsep100 (BiOptic, La Canada Flintridge, CA, USA).

### Transcriptome analysis

RNA samples (biological triplicates in each of two conditions) were run as follows. RNA-seq (2G raw data per sample) was performed by Novogen (Cambridge Science Park, Cambridge, UK) using HiSeq platforms with paired-end 150 bp. Illumina reads have been trimmed for low quality and adapters with fastp (v0.20.0) (Chen et al. 2018) using default settings. rRNA sequences have been removed with bbduk (v38.35) (https://sourceforge.net/projects/bbmap/) using the following parameters: *k* = 31 and ref. = riboKmers.fa.gz. Transcripts from the reference strain of *A*. *muciniphila* (GCF_000020225.1) have been quantified with Kallisto (v0.46.0) (Bray et al. 2016) with a bootstrap value of 100. Transcript abundances were imported using the R/Bioconductor package tximport for differential expression analysis (Soneson et al. 2015). Differential expression analysis has been performed with DESeq2 using the biological replicates for each condition and *p*adj (adjusted *p* values) was calculated using the procedure of Benjamini and Hochberg to avoid false-positive results (Bufe et al. 2019; Love et al. 2014). Differences obtained at the *p*adj < 0.05 level (*n* = 3) were considered significant.

### Membrane transporter inhibitor test

Two membrane transporter inhibitors, orthovanadate (Sigma-Aldrich, S6508; St. Louis, MO, USA) and Phe-Arg β-naphthylamide dihydrochloride (PAβN; Sigma-Aldrich, P4157) known as ABC transporter inhibitor and RND-type transporter inhibitor (Lin and Martinez 2006; Ricci and Piddock 2003), were used for transporter inhibition test. Orthovanadate was dissolved with distilled water and the pH was adjusted to 7.5 (200 mM stock solution). The stock solution was incubated at 90 °C until translucent. PAβN was dissolved in distilled water (0.5 mg/mL stock solution). These stock solutions were sterilized using a 0.22-μm-pore filter. Two hundred microliters of orthovanadate and 100 μL of PAβN were added to medium supplemented with 0.1% ox-bile (final concentrations of inhibitors are 4 mM and 5 μg/mL, respectively). The growth of *A*. *muciniphila* was monitored by OD_600_ measurements.

### Accession number

The RNA-seq data were deposited into the NCBI Sequence Read Archive (SRA) with the BioProject ID PRJNA639650. The BioSample accession numbers “SAMN15311471 to SAMN15311473” and “SAMN15312138 to SAMN15312140” correspond to the data under control and bile acid conditions, respectively.

## Results

### The effect of bile acids on the growth of *A*. *muciniphila*

**Fig. 1:**
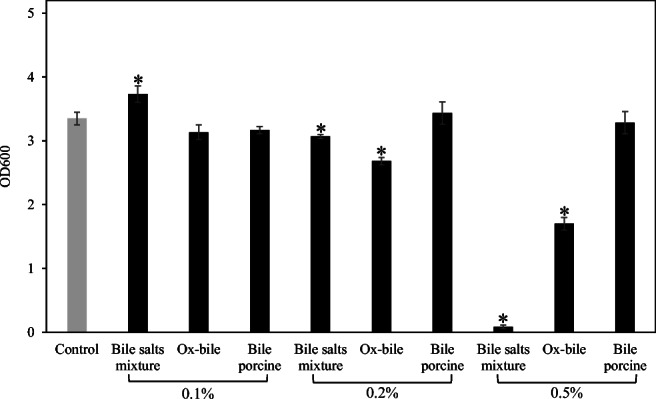
The effect of bile extracts and bile salts mixture on the growth of *A*. *muciniphila*. *A*. *muciniphila* was cultured in the presence of ox-bile, bile salts mixture, and bile porcine (0.1%; 0.2%; 0.5%). After incubation at 37 °C for 48 h, the optical density was measured (OD_600_). These experiments were performed in triplicate (*n* = 3). *Significant difference between control (no treatment with bile acids) and bile-treated groups, Dunnett’s test, *p* < 0.05

To test the tolerance of *A*. *muciniphila* against bile acids, bile salts mixture and major two types of bile acids derived from bovine and porcine were selected (Begley et al. 2002). *A*. *muciniphila* was cultured in the presence of ox-bile, bile salts mixture, and bile porcine at different concentrations (final conc. 0.1-, 0.2-, and 0.5%). After incubation for 48 h, the growth of *A*. *muciniphila* was significantly inhibited at the concentration of 0.2% and 0.5% ox-bile and bile salts mixture (Fig. 1). The growth tended to decrease in cultures containing 0.1% ox-bile (significantly decreased at the 24-h time point, data not shown). On the other hand, the growth of *A*. *muciniphila* significantly increased at bile salt concentrations of 0.1%. Transcriptome analysis and inhibition test of isoprenoid production and membrane transporters were performed at the concentration of 0.1% ox-bile because ox-bile inhibited the growth of *A*. *muciniphila* in a dose-dependent manner and 0.1% ox-bile has a weak inhibitory effect. In addition, the cultures with a concentration of 0.1% bile acids could influence gene expression of *Lactobacillus plantarum* WCFS1 and is within the range of physiological concentration in the gastrointestinal tract (Bron et al. 2004; Hu et al. 2018).

### The effect of individual bile salts on the growth of *A*. *muciniphila*

**Fig. 2:**
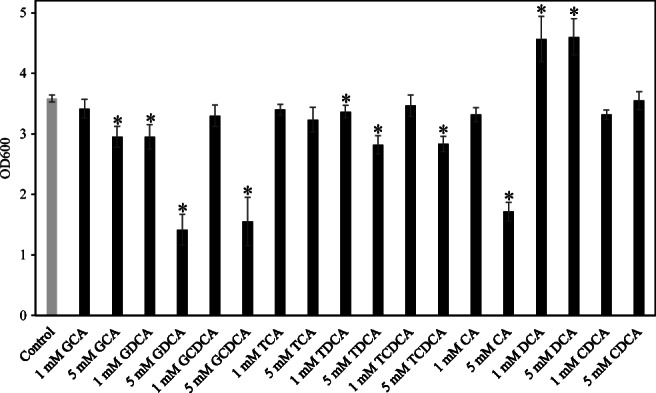
The effect of individual bile salts on the growth of *A*. *muciniphila*. *A*. *muciniphila* was cultured in the presence of individual bile salts at different concentrations (final conc. 1 and 5 mM). After incubation at 37 °C for 48 h, the optical density was measured (OD_600_). These experiments were performed in triplicate (*n* = 3). *Significant difference between control (no treatment with bile salts) and individual bile salt-treated groups, Dunnett’s test, *p* < 0.05

To investigate the effect of individual bile salts, the growth of *A*. *muciniphila* cultured in the presence of 9 bile salts at the different concentrations was assessed (final conc. 1 and 5 mM). Six bile salts (glycocholic acid sodium: GCA, GDCA; sodium glycochenodeoxycholate: GCDCA; sodium taurodeoxycholate hydrate: TDCA; taurochenodeoxycholic acid sodium: TCDCA and CA) were found to inhibit the growth of *A*. *muciniphila* (Fig. 2). Only two of these, GDCA and TDCA, inhibited the growth at a final concentration of 1 mM. There was no significant difference between control and groups treated with other bile salts (TCA and CDCA). Interestingly, the growth of *A*. *muciniphila* significantly increased in the presence of DCA, which is a secondary bile salt, although glycine-conjugated DCA (GDCA) showed the strong inhibition of the growth.

### The effect of squalene synthase inhibitor (zaragozic acid A) on the bile acid tolerance of *A*. *muciniphila*

**Fig. 3:**
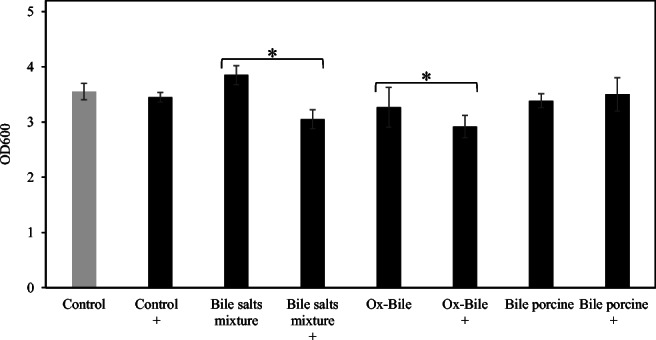
The effect of zaragozic acid A on the tolerance of *A*. *muciniphila* against bile extracts and bile salts mixture. *A*. *muciniphila* was cultured in medium containing 0.1% ox-bile, bile salts mixture, and bile porcine supplemented with or without zaragozic acid A. After incubation at 37 °C for 48 h, the optical density was measured (OD_600_). These experiments were performed in triplicate (*n* = 3). Control means no treatment with bile acids. ^+^Treatment with zaragozic acid A (final concentration 15 μM) *Paired *t* test, *p* < 0.05

Kyoto Encyclopedia of Genes and Genomes (https://www.genome.jp/kegg/) shows *A*. *muciniphila* has isoprenoid biosynthesis genes leading to squalene (EC:2.5.1.21, AMUC_RS02040) and hopanoid (sterol) biosynthesis (EC:5.4.99.17 and EC:4.2.1.129, AMUC_RS02875). In the genome database, 5 genes encoding terpene cyclase/mutase family protein (AMUC_RS06010, AMUC_RS06015, AMUC_RS03775, AMUC_RS03780, and AMUC_RS10605) as well as AMUC_RS02875 exist. ZA, which can inhibit bacterial squalene synthase (Rivas-Marin et al. 2019), was used to investigate the relationship between squalene (precursor of hopanoid) and bile acid tolerance. As a result, the growth of *A*. *muciniphila* cultured with 0.1% bile salts mixture and ox-bile was significantly inhibited upon the addition of ZA (Fig. 3). There is no significant difference in cultures supplemented with 0.1% bile porcine. ZA did not affect the growth of *A*. *muciniphila* cultured without ox-bile (data not shown).

### Change in gene expression in response to ox-bile

**Fig. 4:**
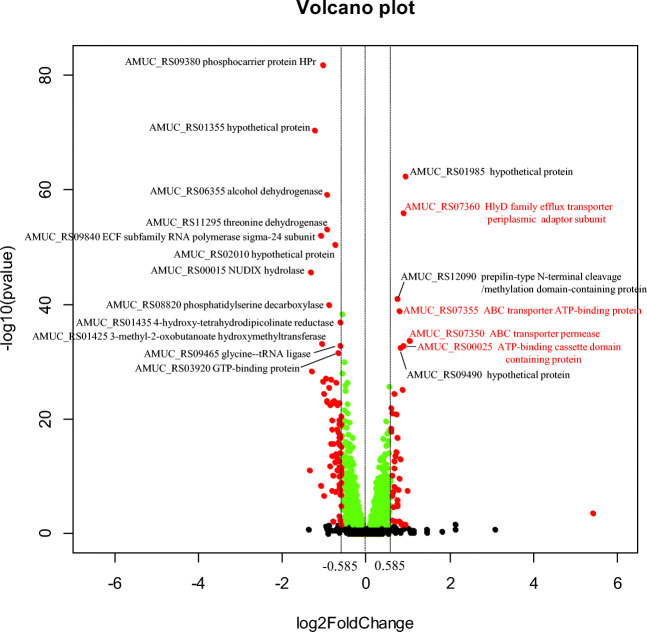
Volcano plot of the ox-bile versus normal condition. Green and red points mean significant change in gene expression under ox-bile condition (*p*adj < 0.05). The red point means log2 fold changes cut-off < − 0.585 and > 0.585. Volcano plot was described by R 3.6.1. The data corresponding to significant differences in gene expression under ox-bile conditions can be found in Supplemental Table S1. These experiments were performed in triplicate (*n* = 3)

The change in gene expression in cultures with and without 0.1% ox-bile was determined by DESeq and visualized using a volcano plot (Fig. 4). There were 1008 significant differentially expressed genes (green and red, *p*adj < 0.05) which contained 454 upregulated genes and 554 downregulated genes (all genes are listed in Supplementary Table S1).

**Fig. 5:**
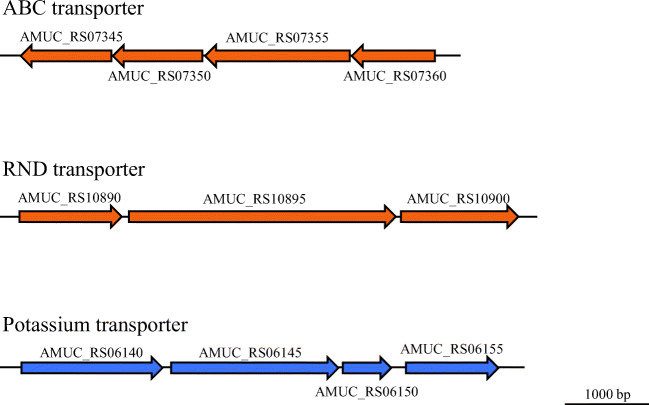
Gene clusters up- and downregulated under ox-bile condition. The orange and blue arrows show the up- and downregulated gene clusters, respectively

Figure 4 (red point) and Table 1 show the upregulated and downregulated genes under ox-bile condition (*p*adj < 0.05, log2 fold changes with cut-off at < − 0.585 and > 0.585). Thirty-eight genes were upregulated in the cultures supplemented with ox-bile (Table 1). The gene expression of the ABC transporter systems (AMUC_RS07350, AMUC_RS00025, AMUC_RS07355, and AMUC_RS07345) and ABC transporter–associated HlyD family efflux transporter periplasmic adaptor subunit (AMUC_RS07360) was significantly upregulated. Four genes (AMUC_RS07345 to AMUC_RS07360) are considered to form a gene cluster of ABC transporter–associated genes (Fig. 5). Efflux RND transporter periplasmic adaptor subunit (AMUC_RS10890) and two genes, downstream of AMUC_RS10890, were slightly but significantly upregulated (*p*adj < 0.05, log2 fold change of AMUC_RS10895 and AMUC_RS10900 was 0.495 and 0.396, respectively, Supplementary Table S1). In addition, PEP-CTERM domain protein (AMUC_RS10910), which is a membrane protein, was also upregulated. The DnaK gene (AMUC_RS07510), which is the stress response gene known to encode a major stress-inducible chaperone (LaRossa and Van Dyk 1991), was also significantly upregulated. Concerning hopanoid biosynthesis–related genes, four genes encoding terpene cyclase/mutase family protein (AMUC_RS03775, AMUC_RS06010, AMUC_RS06015, and AM UC_RS03775) were slightly but significantly upregulated (*p*adj < 0.05, log2 fold change = 0.211, 0.270, 0.260, and 0.210, Supplementary Table S1). The gene expression of bile acid:sodium symporter (Amuc_0139) was not found to be significantly different in these conditions.

**Table 1:** Differential gene expression in response to ox-bile

Locus_tag	Log2 fold change	*p*adj	ID	Product	Protein_ID
Upregulated genes					
AMUC_RS04540	5.426	0.00106	gene905	Hypothetical protein	WP_042447787.1
AMUC_RS07350	1.032	3.12E−32	gene1461	ABC transporter permease	WP_012420419.1
AMUC_RS04835	0.981	2.80E−07	gene964	Hypothetical protein	WP_042447818.1
AMUC_RS01985	0.938	2.64E−60	gene395	Hypothetical protein	WP_012419409.1
AMUC_RS00145	0.930	0.044701	gene28	Hypothetical protein	WP_042447393.1
AMUC_RS00025	0.901	2.25E−31	gene4	ATP-binding cassette domain-containing protein	WP_012419065.1
AMUC_RS07360	0.901	5.31E−54	gene1463	HlyD family efflux transporter periplasmic adaptor subunit	WP_012420421.1
AMUC_RS00090	0.865	4.54E−24	gene17	Hypothetical protein	WP_042447385.1
AMUC_RS11530	0.838	0.040573	gene2300	Hypothetical protein	WP_012421183.1
AMUC_RS02315	0.833	0.044265	gene461	Hypothetical protein	WP_042447591.1
AMUC_RS09490	0.809	3.43E−31	gene1890	Hypothetical protein	WP_012420811.1
AMUC_RS03250	0.809	1.76E−12	gene648	Phospholipid/glycerol acyltransferase	WP_012419647.1
AMUC_RS03405	0.805	2.22E−09	gene679	Peptidase M60	WP_012419679.1
AMUC_RS07355	0.793	3.10E−37	gene1462	ABC transporter ATP-binding protein	WP_012420420.1
AMUC_RS03410	0.792	0.019798	gene680	Hypothetical protein	WP_042447676.1
AMUC_RS04655	0.770	2.01E−07	gene928	Hypothetical protein	
AMUC_RS12090	0.749	2.69E−39	gene1685	Prepilin-type N-terminal cleavage/methylation domain–containing protein	WP_012420618.1
AMUC_RS04775	0.749	7.39E−05	gene952	Hypothetical protein	WP_042447805.1
AMUC_RS02165	0.743	5.90E−16	gene431	tRNA pseudouridine synthase A	WP_012419443.1
AMUC_RS04970	0.736	3.86E−05	gene992	Glycosyl hydrolase family 109 protein 2	WP_012419967.1
AMUC_RS07595	0.735	8.01E−06	gene1509	Hypothetical protein	WP_042448077.1
AMUC_RS07345	0.731	5.04E−20	gene1460	Antibiotic ABC transporter permease	WP_012420418.1
AMUC_RS08910	0.729	1.17E−13	gene1774	Hypothetical protein	WP_042448215.1
AMUC_RS05455	0.707	5.40E−13	gene1087	Hypothetical protein	WP_012420058.1
AMUC_RS05825	0.690	0.019118	gene1159	Hypothetical protein	WP_042447906.1
AMUC_RS07530	0.682	4.59E−08	gene1496	Phosphate/sulfate permease	WP_012420448.1
AMUC_RS09690	0.673	2.19E−23	gene1930	Hypothetical protein	WP_012420848.1
AMUC_RS10910	0.662	3.96E−12	gene2175	PEP-CTERM domain protein	WP_012421069.1
AMUC_RS10505	0.660	5.80E−11	gene2095	Hypothetical protein	WP_012420995.1
AMUC_RS07510	0.652	3.72E−07	gene1492	Molecular chaperone DnaK	WP_012420444.1
AMUC_RS11765	0.648	0.000103	gene410	Hypothetical protein	WP_052294421.1
AMUC_RS09570	0.629	0.010839	gene1906	Hypothetical protein	WP_042448291.1
AMUC_RS07260	0.628	1.43E−06	gene1443	Hypothetical protein	WP_042448027.1
AMUC_RS02540	0.620	3.91E−20	gene506	PDZ/DHR/GLGF domain–containing protein	WP_012419514.1
AMUC_RS06360	0.616	9.94E−10	gene1266	Holliday junction DNA helicase	WP_012420231.1
AMUC_RS00050	0.597	1.24E−17	gene9	Pseudouridine synthase	WP_012419070.1
AMUC_RS01000	0.594	5.33E−17	gene198	Hypothetical protein	WP_012419233.1
AMUC_RS10890	0.589	5.84E−21	gene2171	Efflux RND transporter periplasmic adaptor subunit	WP_051729712.1
**Downregulated genes**					
AMUC_RS00360	− 1.343	1.18E−10	gene72	Membrane protein	WP_042448508.1
AMUC_RS00015	− 1.317	5.85E−44	gene2	NUDIX hydrolase	WP_012419063.1
AMUC_RS07395	− 1.303	3.58E−27	gene1470	Short-chain dehydrogenase/reductase SDR	WP_012420428.1
AMUC_RS01355	− 1.225	5.07E−68	gene268	Hypothetical protein	
AMUC_RS06150	− 1.095	3.31E−08	gene1224	Potassium transporter KtrB	WP_012420193.1
AMUC_RS09840	− 1.084	2.88E−50	gene1961	ECF subfamily RNA polymerase sigma-24 subunit	WP_012420873.1
AMUC_RS01425	− 1.051	1.07E−31	gene282	3-Methyl-2-oxobutanoate hydroxymethyltransferase	WP_012419313.1
AMUC_RS01260	− 1.043	2.12E−25	gene249	Hypothetical protein	WP_042447516.1
AMUC_RS09380	− 1.039	3.00E−79	gene1868	Phosphocarrier protein Hpr	WP_012420789.1
AMUC_RS06145	− 1.017	1.45E−06	gene1223	Potassium-transporting ATPase subunit B	WP_012420192.1
AMUC_RS02660	− 1.006	2.49E−23	gene530	50S ribosomal protein L21	WP_012419537.1
AMUC_RS06345	− 0.957	7.49E−26	gene1263	50S ribosomal protein L28	WP_012420229.1
AMUC_RS08450	− 0.948	4.78E−22	gene1682	Nucleoside-diphosphate kinase	WP_012420615.1
AMUC_RS09030	− 0.941	3.91E−22	gene1798	Hypothetical protein	WP_042448236.1
AMUC_RS11295	− 0.932	2.99E−51	gene2253	Threonine dehydrogenase	WP_012421139.1
AMUC_RS06355	− 0.926	3.22E−57	gene1265	Alcohol dehydrogenase	WP_012420230.1
AMUC_RS02895	− 0.896	2.05E−24	gene577	Glutamate 5-kinase	WP_012419582.1
AMUC_RS08820	− 0.894	2.28E−38	gene1756	Phosphatidylserine decarboxylase	WP_012420686.1
AMUC_RS05935	− 0.861	1.41E−21	gene1181	ATP phosphoribosyltransferase	WP_012420148.1
AMUC_RS09680	− 0.858	3.10E−11	gene1928	Hypothetical protein	WP_042448307.1
AMUC_RS01595	− 0.836	6.37E−15	gene316	Ribonuclease HIII	WP_042448620.1
AMUC_RS02580	− 0.834	1.11E−25	gene514	N-Acetyltransferase GCN5	WP_012419522.1
AMUC_RS03790	− 0.814	2.43E−17	gene755	Beta-glucanase	WP_012419750.1
AMUC_RS06035	− 0.812	5.83E−13	gene1201	Hypothetical protein	WP_012420169.1
AMUC_RS05250	− 0.807	7.34E−19	gene1048	Hypothetical protein	WP_012420024.1
AMUC_RS06140	− 0.804	3.10E−07	gene1222	Potassium-transporting ATPase subunit KdpA	WP_022196803.1
AMUC_RS05000	− 0.795	8.76E−10	gene998	50S ribosomal protein L5	WP_012419973.1
AMUC_RS01360	− 0.793	5.73E−22	gene269	tRNA (guanine(37)-N(1))-methyltransferase	WP_042448603.1
AMUC_RS04320	− 0.784	0.017267	gene861	Cupin	WP_012419849.1
AMUC_RS10155	− 0.779	6.39E−15	gene2026	Nitrogen-fixing protein NifU	WP_012420930.1
AMUC_RS00740	− 0.779	7.93E−22	gene147	Transposase	WP_012419186.1
AMUC_RS04995	− 0.769	4.30E−22	gene997	50S ribosomal protein L24	WP_012419972.1
AMUC_RS01750	− 0.746	5.21E−12	gene347	30S ribosomal protein S12	WP_012419365.1
AMUC_RS08815	− 0.744	4.18E−07	gene1755	Secretion protein	WP_012420685.1
AMUC_RS02670	− 0.739	4.76E−12	gene532	Transcriptional repressor	WP_012419539.1
AMUC_RS02010	− 0.735	1.06E−48	gene400	Hypothetical protein	WP_042447573.1
AMUC_RS05195	− 0.716	2.85E−13	gene1037	Hypothetical protein	WP_051729423.1
AMUC_RS02675	− 0.716	3.12E−25	gene533	Fe-S cluster assembly ATPase SufC	WP_035196050.1
AMUC_RS11880	− 0.704	1.29E−10	gene875	Hypothetical protein	WP_052294442.1
AMUC_RS05795	− 0.701	1.23E−12	gene1153	23S rRNA (guanosine(2251)-2′-O)-methyltransferase RlmB	WP_012420121.1
AMUC_RS09135	− 0.692	2.57E−17	gene1819	Recombinase RecQ	WP_012420743.1
AMUC_RS10835	− 0.683	1.69E−21	gene2160	Dihydrofolate reductase	WP_012421055.1
AMUC_RS07650	− 0.661	6.98E−17	gene1521	50S ribosomal protein L31	WP_035196558.1
AMUC_RS06690	− 0.659	3.27E−15	gene1330	GDP-mannose 4%2C6-dehydratase	WP_012420289.1
AMUC_RS03920	− 0.658	2.58E−30	gene781	GTP-binding protein	WP_012419774.1
AMUC_RS01615	− 0.655	3.21E−18	gene320	Hypothetical protein	WP_012419341.1
AMUC_RS01735	− 0.655	2.19E−08	gene344	30S ribosomal protein S10	WP_012419362.1
AMUC_RS00220	− 0.654	1.37E−07	gene43	N-Acetyltransferase GCN5	WP_012419101.1
AMUC_RS06205	− 0.645	3.70E−11	gene1235	Amino acid lyase	WP_012420202.1
AMUC_RS09155	− 0.644	1.09E−09	gene1823	Succinyl-CoA ligase subunit beta	WP_012420746.1
AMUC_RS06540	− 0.644	6.88E−15	gene1301	Hypothetical protein	WP_042447975.1
AMUC_RS05005	− 0.643	4.22E−16	gene999	30S ribosomal protein S8	WP_012419974.1
AMUC_RS02380	− 0.640	5.42E−19	gene474	CinA-like protein	WP_012419483.1
AMUC_RS03785	− 0.638	4.04E−12	gene754	Beta-glucanase	WP_012419749.1
AMUC_RS10055	− 0.635	9.20E−22	gene2005	MBL fold metallo-hydrolase	WP_012420911.1
AMUC_RS05010	− 0.634	0.002924	gene1000	50S ribosomal protein L6	WP_012419975.1
AMUC_RS09395	− 0.632	5.24E−16	gene1871	ABC transporter ATP-binding protein	WP_012420792.1
AMUC_RS05015	− 0.630	1.05E−06	gene1001	50S ribosomal protein L18	WP_012419976.1
AMUC_RS09465	− 0.623	2.25E−31	gene1885	Glycine-tRNA ligase	WP_012420806.1
AMUC_RS01435	− 0.623	1.77E−35	gene284	4-Hydroxy-tetrahydrodipicolinate reductase	WP_012419315.1
AMUC_RS01170	− 0.622	1.77E−18	gene231	Glucose-1-phosphate thymidylyltransferase	WP_012419267.1
AMUC_RS11245	− 0.621	2.42E−16	gene2243	Malate dehydrogenase	WP_012421129.1
AMUC_RS01745	− 0.620	0.010316	gene346	30S ribosomal protein S7	WP_012419364.1
AMUC_RS01390	− 0.615	1.50E−07	gene275	GNAT family acetyltransferase	WP_012419307.1
AMUC_RS04460	− 0.614	1.47E−13	gene889	DNA-binding response regulator	WP_012419876.1
AMUC_RS11080	− 0.614	3.24E−11	gene2210	Hypothetical protein	WP_012421098.1
AMUC_RS07515	− 0.605	0.04402	gene1493	Molecular chaperone GroES	WP_012420445.1
AMUC_RS01690	− 0.604	1.05E−06	gene335	50S ribosomal protein L16	WP_012419353.1
AMUC_RS10500	− 0.604	7.83E−11	gene2094	Thioredoxin	WP_012420994.1
AMUC_RS08060	− 0.602	1.20E−08	gene1603	DNA-directed RNA polymerase subunit alpha	WP_012420541.1
AMUC_RS06385	− 0.599	1.46E−14	gene1271	Hypothetical protein	WP_031930834.1
AMUC_RS10950	− 0.598	1.14E−19	gene2183	Glutamate dehydrogenase	WP_012421075.1
AMUC_RS07810	− 0.597	3.84E-11	gene1553	Phosphoribosylformimino-5-aminoimidazole carboxamide ribotide isomerase	WP_012420494.1
AMUC_RS09600	− 0.593	6.92E−05	gene1912	Fe–S center ferredoxin	WP_012420831.1
AMUC_RS01955	− 0.593	2.32E−10	gene389	ATP-binding protein	WP_012419404.1
AMUC_RS08145	− 0.590	3.01E−18	gene1620	Type III restriction endonuclease subunit R	WP_012420558.1
AMUC_RS03260	− 0.585	4.81E−10	gene650	Flavin reductase	WP_042448704.1

Log2 fold change = ox-bile/control condition (*n* = 3; the experiment was performed in triplicate)

Padj was calculated using the procedure of Benjamini and Hochberg. The upregulated and downregulated genes under ox-bile condition (*p*adj < 0.05, log2 fold changes with cut-off at < − 0.585 and > 0.585) were listed

Furthermore, 77 genes were downregulated in the presence of ox-bile. In contrast to the upregulation of ABC transporters, the potassium transport system (AMUC_RS06145 and AMUC_RS06150) was significantly downregulated. Neighboring genes (AMUC_RS06140 encoding potassium-transporting ATPase subunit KdpA and AMUC_RS06155 encoding osmosensitive K channel His kinase sensor), part of the gene cluster of the potassium transport system, were also slightly downregulated (*p*adj < 0.05, − 0.804, and − 0.453 fold change, respectively). Some enzymes belonging to dehydrogenase, hydrogenase, decarboxylase, ligase, and reductase were also downregulated (Table 1). Although the stress protein DnaK gene was upregulated, the GroES gene was downregulated by ox-bile. In addition, the gene encoding short-chain dehydrogenase/reductase SDR (AMUC_RS07395), which is a member of steroid degradation enzymes (Ji et al. 2014), was downregulated under ox-bile condition.

### The effect of membrane transporter inhibitor on ox-bile tolerance

**Fig. 6:**
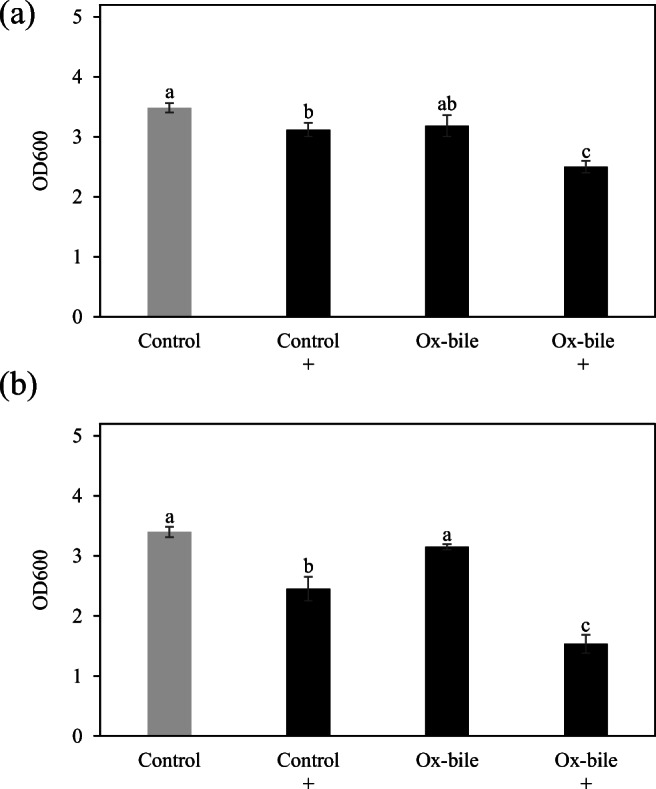
The effect of transporter inhibitor on the tolerance of *A*. *muciniphila* against ox-bile. *A*. *muciniphila* was cultured in medium containing 0.1% ox-bile supplemented with or without 4 mM orthovanadate (**a**) or 5 μg/mL PaβN (**b**). After incubation at 37 °C for 48 h, the optical density was measured (OD_600_). These experiments were performed in triplicate (*n* = 3). Control means no treatment with ox-bile. ^+^Treatment with an inhibitor. Different letters denote significant differences (Tukey’s HSD test, *p* < 0.05)

Transcriptome analysis showed the gene expression of HlyD-ABC and RND type transporters was upregulated under ox-bile condition. To investigate whether these transporters are related to bile acid tolerance in *A*. *muciniphila*, an inhibitor test using orthovanadate and PAβN (Phe-Arg β-naphthylamide dihydrochloride) was performed. Although orthovanadate inhibited the growth of *A*. *muciniphila* under control condition, the highest inhibition was observed under ox-bile with orthovanadate condition (Fig. 6a). In addition, PAβN, which is an RND efflux pump inhibitor, also reduced the ox-bile tolerance of *A*. *muciniphila* (Fig. 6b).

## Discussion

Bile acids can affect the microbial composition in the gut (Wahlstrom et al. 2016). *A*. *muciniphila* is an interesting gut bacterium correlated with host health. Herein, the growth and change in gene expression of *A*. *muciniphila* in response to bile acids were investigated. All tested bile extracts except for bile extract from porcine inhibited the growth of *A*. *muciniphila* (Fig. 1). The difference in the phospholipid and the hydroxylation of glycine and tauroconjugate composition between bovine and porcine bile was reported (Coleman et al. 1979; Farthing et al. 1985). A different bile acid or phospholipid composition may cause a non-inhibitory effect of bile extract from porcine although the difference of this mechanism is not known. Primary bile acids such as CA, GCA, and GCDCA showed an inhibitory effect against *A*. *muciniphila* (Fig. 2). Interestingly, this study also showed the increased growth of *A*. *muciniphila* cultured with secondary bile acid DCA (Fig. 2) or a low concentration of bile salts mixture (Sigma) consisting of CA and DCA (in a ratio of 1:1) (Fig. 1). On the other hand, a high concentration of bile salts mixture strongly inhibited the growth of *A*. *muciniphila* (Fig. 1). Our previous review showed that *A*. *muciniphila* is abundantly present in the large intestine (Geerlings et al. 2018), where primary bile acids can be converted to secondary bile acids (Foley et al. 2019). The concentration of CA or ratio of DCA to CA may affect the growth of *A*. *muciniphila* in the gut. Another report demonstrated that DCA-induced MUC2 protein expression of human colon carcinoma cells leads to mucin production which is a carbon source of *A*. *muciniphila* (Song et al. 2005). These results indicate that DCA is considered to be an important factor for *A*. *muciniphila* to persist in the gut. Several genera such as *Rhodococcus* and *Mycobacterium* have meta-cleavage dioxygenases to degrade DCA (Merino et al. 2013). However, no gene encoding a meta-cleavage dioxygenase was observed in the genome of *A*. *muciniphila*. Ursodeoxycholic acid could also increase the cell number of *A*. *muciniphila* in mice (Van den Bossche et al. 2017). Another report showed that the increase of bile acids (especially, cholic acid) could reduce the population of *A*. *muciniphila* in mice (Zheng et al. 2017). These results implied that a balance of bile acids plays an important role for the growth of *A*. *muciniphila* in the gut. Additional experiments are needed to clarify the relationship between secondary bile acids and the metabolism of *A*. *muciniphila*.

Squalene synthase inhibitor ZA inhibited the growth of *A*. *muciniphila* in the presence of bile extracts and mixture (Fig. 3). Squalene is the precursor of hopanoid which is required for bile acid tolerance and other stress conditions in *Rhodopseudomonas palustris* (Welander et al. 2009). This report indicated that a lack of hopanoids results in increased membrane permeability and could decrease bile acid tolerance. Transcriptome analysis also showed that terpene cyclase/mutase family proteins (AMUC_RS03775, AMUC_RS06010, AMUC_RS06015, and AMUC_RS03775), which are considered to be associated with hopanoid production, were upregulated in the presence of ox-bile. These results indicate that hopanoid production associated with membrane permeability could contribute to bile acid tolerance in *A*. *muciniphila*.

The mechanism in stress response to bile acids was investigated by analyzing the transcriptional response of *A*. *muciniphila* in the presence of ox-bile. A gene cluster of the ABC transporter system shown in Fig. 5 was significantly upregulated. ABC transporters are divided into several groups with different characteristics associated with the uptake of nutrients and export of drugs (Locher 2016). ABC transporter BmrAB, comprising of 652– and 671–amino acid proteins, is required for bile acid tolerance in *Bifidobacterium longum* BBMN68 (Xu et al. 2019). The amino acid sequence of BmrA (652 aa, BBMN68_1797) in *B*. *longum* is similar to the ABC transporter ATP-binding protein AMUC_RS00905 (34% identity; 54% similarity, 594 aa). BmrB (671 aa, BBMN68_1798) is similar with ABC transporter ATP-binding protein AMUC_RS00910 (47% identity; 64% similarity, 616 aa). Their genes are different in comparison to the upregulated ABC transporter genes in Fig. 5. In addition, the ABC transporter gene cluster of *A*. *muciniphila* contains the gene for HlyD (AMUC_RS07360) known as a periplasmic adaptor protein (Symmons et al. 2015). *A*. *muciniphila* may use ABC-type multidrug transport systems different from *Bifidobacterium* to improve the tolerance to bile acids. A gene encoding an efflux RND transporter periplasmic adaptor subunit (AMUC_RS10890) and a gene encoding an efflux RND transporter permease subunit (AMUC_RS10895) were also upregulated in the presence of ox-bile. RND (resistance-nodulation-division) is known as a part of a transporter system (Symmons et al. 2015). RND transporters as well as ABC transporters are considered to be bile acid response genes in *Campylobacterales* (Okoli et al. 2007). These results imply that both types of transporter systems (RND and ABC) may be bile response genes required for bile acid tolerance in *A*. *muciniphila*.

Both membrane transporter (ABC and RND type) inhibitory tests using orthovanadate and PAβN supported the transcriptome analysis (Fig. 6). These transporter inhibitors were also used for inhibitory test of transporters in an obligately anaerobic gut bacteria *Bacteroides fragilis* (Ricci and Piddock 2003). Alternatively, these inhibitors also slightly reduced the growth of *A*. *muciniphila* under the control condition. Orthovanadate might inhibit other ABC transporters required for growth or cause a oxidative stress (Minasi and Willsky 1991; Schneider and Hunke 1998). PAβN also might inhibit an efflux pump required for growth or cause a weak membrane-destabilization although it is known as an efflux pump inhibitor used for bile tolerance tests (Lin and Martinez 2006; Misra et al. 2015; Sannasiddappa et al. 2015) as well as antibiotic resistance tests (Chitsaz et al. 2019). However, transporters could be strongly related to bile acid tolerance in *A*. *muciniphila* because the addition of transporter inhibitor showed the strongest growth inhibition under bile acid condition. Alternatively, transporter inhibitors, which are originally developed as an inhibition of antibiotic resistance bacteria (Shriram et al. 2018), strongly inhibited the growth of *A*. *muciniphila* under bile acid condition. These results imply that the impact of transporter inhibitors on commensal gut microbiota such as *A*. *muciniphila* is needed for our health.

Upregulation of the gene encoding a PEP-CTERM domain protein (AMUC_RS10910) was also observed. This protein is related to exopolysaccharide biosynthesis (Haft et al. 2006). Other exopolysaccharide-associated genes (Supplemental Table S1) and the gene for capsular polysaccharide biosynthesis protein (AMUC_RS07555, AMUC_RS11095) and polysaccharide export protein (AMUC_RS07560) were slightly but significantly downregulated (log2 fold change = − 0.142, − 0.217, and − 0.258, respectively). In contrast, the gene for polysaccharide deacetylase (AMUC_RS08035), which is associated with the hydrolysis of either the N-linked acetyl group from GlcNAc or *O*-linked acetyl groups from *O*-acetylxylose residues (Balomenou et al. 2013), was significantly upregulated (log2 fold change = 0.237). These results imply that EPS (exopolysaccharides) modification leading to a change in membrane composition rather than biosynthesis could occur in response to ox-bile and contribute to bile acid tolerance.

On the other hand, a gene cluster containing a potassium transporter was significantly downregulated in the presence of ox-bile. Potassium ions are abundant inside the cells and regulated by the external K^+^ concentration (Kuo et al. 2005). The K^+^ transport operon is upregulated by K^+^ limitation and high osmolarity and downregulated by high concentration of K+ in *Salmonella typhimurium* (Frymier et al. 1997). Membrane stress or disturbance of potassium balance caused by ox-bile–associated membrane damage may lead to downregulation of a K^+^ transporter. In addition, the genes for a membrane protein (AMUC_RS00360), NUDIX hydrolase (AMUC_RS00015), and short-chain dehydrogenase/reductase SDR (AMUC_RS07395) were the top 3 highly downregulated genes under ox-bile condition (Table 1). One of the short-chain dehydrogenase/reductase (SDR) is known as 7α-hydroxysteroid dehydrogenase with a N-terminal Gly-X-X-X-Gly-X-Gly and a Tyr-X-X-X-Lys segment, which may be related to steroid degradation such as bile acid (Ji et al. 2014). This result implies there may be bile acid metabolism using SDR in *A*. *muciniphila* after transport of bile acids like in *Bifidobacterium* and *Clostridium* with a 7α/7β-dehydroxylation pathway (Ridlon et al. 2016).

**Fig. 7:**
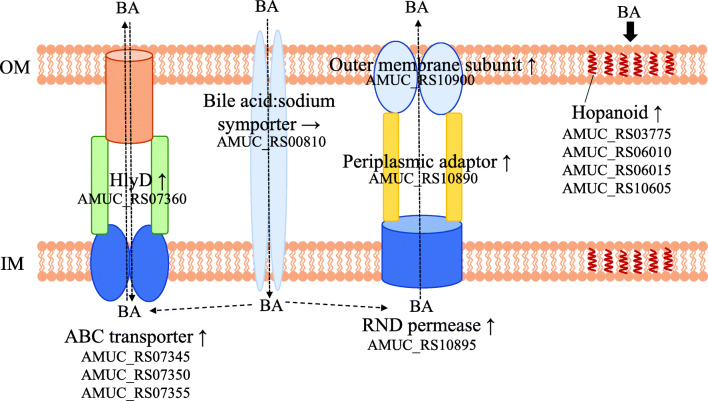
Putative bile acid response system in *A*. *muciniphila*. After an ox-bile exposure, the expression level of genes encoding ABC transporter (AMUC_RS07345 to AMUC_RS07360), RND type transporter (AMUC_RS10890 to AMUC_RS10900), and hopanoid biosynthesis (AMUC_RS03775, AMUC_RS06010, AMUC_RS06015, and AMUC_RS10605) was upregulated. The inhibitors against transporters and hopanoid biosynthesis reduced the tolerance against ox-bile. The change in membrane transporters and membrane composition caused by hopanoid production could contribute to bile tolerance in *A*. *muciniphila* like other bacteria. No change in gene expression of AMUC_RS00810 encoding bile acid:sodium symporter was observed. Bile acid (BA), ↑ upregulated gene, ↓ downregulated gene, → no change in gene expression

Our results suggest that membrane-associated molecules such as isoprenoids (squalene and hopanoids) and transporters could be important factors in bile acid tolerance (Fig. 7). The change in membrane composition caused by hopanoid production could protect cell membrane from bile acids. In addition, although no change in gene expression of AMUC_RS00810 encoding bile acid:sodium symporter was observed, this symporter and other transporter systems (ABC and RND type) may contribute to transport of bile acids and its metabolism like in other bacteria (Lin et al. 2003; Locher 2016; Ruiz et al. 2013). Further characterization on a phenotypic level will help us understand the bile acid response mechanism of *A*. *muciniphila*. The change in gene expression and physiology of *A*. *muciniphila* in response to bile acids can provide novel information on bacterial persistence in the gut. Modulation of secondary bile acids could be a novel target for increasing the growth of *A*. *muciniphila* in the gut and preventing metabolic syndrome and gut disease.

## Electronic supplementary material

ESM 1 (PDF 709 kb)

## Data Availability

*A*. *muciniphila* used in this study is MucT (DSM 22959).
