# Supplementary material for: The effect of bile acids on the growth and global gene expression profiles in Akkermansia muciniphila
Source: Appl Microbiol Biotechnol. 2020 Nov 7;104(24):10641–53. doi: 10.1007/s00253-020-10976-3 (PMC7671984; doi:10.1007/s00253-020-10976-3)
Supplement: Supplementary file 1 — (PDF 709 kb) [file 253_2020_10976_MOESM1_ESM.pdf]

Journal: Applied and Microbiology and Biotechnology

Manuscript Title: The effect of bile acids on the growth and global gene expression profiles in *Akkermansia muciniphila*

Author name: Tatsuro Hagi [1,2]*, Sharon Y. Geerlings [1], Bart Nijsse [3], Clara Belzer [1]*

Affiliation of the authors: [1]Laboratory of Microbiology, Wageningen University and Research, 6708 WE Wageningen, The Netherlands, [2]Animal Products Research Division, Institute of Livestock and Grassland Science, National Agriculture and Food Research Organization (NARO), Ibaraki 305-0901 Japan, [3]Systems and Synthetic Biology, Wageningen University and Research, 6708 WE Wageningen, The Netherlands

The e-mail address and telephone number of the corresponding authors

Clara Belzer: E-mail: clara.belzer@wur.nl, Phone: +31 317 482 795

Tatsuro Hagi: E-mail: thagi@affrc.go.jp, Phone: +81 29 838 8685

Supplemental Table S1 Significant differential expression of genes under ox-bile condition

Locus_tag	log2Fold Change	padj	ID	Product	Protein_ID
**Upregulated genes**					
AMUC_RS04540	5.426	0.00106	gene905	Hypothetical protein	WP_042447787.1
AMUC_RS07350	1.032	3.12E-32	gene1461	ABC transporter permease	WP_012420419.1
AMUC_RS04835	0.981	2.8E-07	gene964	Hypothetical protein	WP_042447818.1
AMUC_RS01985	0.938	2.64E-60	gene395	Hypothetical protein	WP_012419409.1
AMUC_RS00145	0.930	0.044701	gene28	Hypothetical protein	WP_042447393.1
AMUC_RS00025	0.901	2.25E-31	gene4	ATP-binding cassette domain-containing protein	WP_012419065.1
AMUC_RS07360	0.901	5.31E-54	gene1463	HlyD family efflux transporter periplasmic adaptor subunit	WP_012420421.1
AMUC_RS00090	0.865	4.54E-24	gene17	Hypothetical protein	WP_042447385.1
AMUC_RS11530	0.838	0.040573	gene2300	Hypothetical protein	WP_012421183.1
AMUC_RS02315	0.833	0.044265	gene461	Hypothetical protein	WP_042447591.1
AMUC_RS09490	0.809	3.43E-31	gene1890	Hypothetical protein	WP_012420811.1
AMUC_RS03250	0.809	1.76E-12	gene648	Phospholipid/glycerol acyltransferase	WP_012419647.1
AMUC_RS03405	0.805	2.22E-09	gene679	Peptidase M60	WP_012419679.1
AMUC_RS07355	0.793	3.1E-37	gene1462	ABC transporter ATP-binding protein	WP_012420420.1
AMUC_RS03410	0.792	0.019798	gene680	Hypothetical protein	WP_042447676.1
AMUC_RS04655	0.770	2.01E-07	gene928	Hypothetical protein	
AMUC_RS12090	0.749	2.69E-39	gene1685	Prepilin-type N-terminal cleavage/methylation domain-containing protein	WP_012420618.1
AMUC_RS04775	0.749	7.39E-05	gene952	Hypothetical protein	WP_042447805.1
AMUC_RS02165	0.743	5.9E-16	gene431	Trna pseudouridine synthase A	WP_012419443.1
AMUC_RS04970	0.736	3.86E-05	gene992	Glycosyl hydrolase family 109 protein 2	WP_012419967.1
AMUC_RS07595	0.735	8.01E-06	gene1509	Hypothetical protein	WP_042448077.1
AMUC_RS07345	0.731	5.04E-20	gene1460	Antibiotic ABC transporter permease	WP_012420418.1
AMUC_RS08910	0.729	1.17E-13	gene1774	Hypothetical protein	WP_042448215.1
AMUC_RS05455	0.707	5.4E-13	gene1087	Hypothetical protein	WP_012420058.1
AMUC_RS05825	0.690	0.019118	gene1159	Hypothetical protein	WP_042447906.1
AMUC_RS07530	0.682	4.59E-08	gene1496	Phosphate/sulfate permease	WP_012420448.1
AMUC_RS09690	0.673	2.19E-23	gene1930	Hypothetical protein	WP_012420848.1
AMUC_RS10910	0.662	3.96E-12	gene2175	PEP-CTERM domain protein	WP_012421069.1
AMUC_RS10505	0.660	5.8E-11	gene2095	Hypothetical protein	WP_012420995.1
AMUC_RS07510	0.652	3.72E-07	gene1492	Molecular chaperone dnak	WP_012420444.1
AMUC_RS11765	0.648	0.000103	gene410	Hypothetical protein	WP_052294421.1
AMUC_RS09570	0.629	0.010839	gene1906	Hypothetical protein	WP_042448291.1
AMUC_RS07260	0.628	1.43E-06	gene1443	Hypothetical protein	WP_042448027.1
AMUC_RS02540	0.620	3.91E-20	gene506	PDZ/DHR/GLGF domain-containing protein	WP_012419514.1

AMUC_RS06360	0.616	9.94E-10	gene1266	Holliday junction DNA helicase	WP_012420231.1
AMUC_RS00050	0.597	1.24E-17	gene9	Pseudouridine synthase	WP_012419070.1
AMUC_RS01000	0.594	5.33E-17	gene198	Hypothetical protein	WP_012419233.1
AMUC_RS10890	0.589	5.84E-21	gene2171	Efflux RND transporter periplasmic adaptor subunit	WP_051729712.1
AMUC_RS10150	0.584	8.17E-09	gene2025	Metallophosphoesterase	WP_012420929.1
AMUC_RS08510	0.578	2.22E-09	gene1694	Superoxide dismutase	WP_012420627.1
AMUC_RS06840	0.564	5.21E-05	gene1360	Hypothetical protein	WP_012420316.1
AMUC_RS06225	0.552	1.38E-09	gene1239	Hypothetical protein	WP_052294457.1
AMUC_RS06000	0.551	1.28E-24	gene1194	Hypothetical protein	WP_012420162.1
AMUC_RS04810	0.551	0.000522	gene959	Mexx family efflux pump subunit	WP_012419937.1
AMUC_RS01965	0.549	3.2E-17	gene391	1,4-dihydroxy-2-naphthoyl-CoA synthase	WP_012419405.1
AMUC_RS05335	0.545	1.75E-13	gene1064	Hypothetical protein	WP_012420036.1
AMUC_RS01130	0.542	3.51E-06	gene223	Methylmalonyl-coa epimerase	WP_012419259.1
AMUC_RS00540	0.535	0.006843	gene108	Hypothetical protein	WP_042447447.1
AMUC_RS09120	0.531	0.044527	gene1816	Hypothetical protein	WP_012420741.1
AMUC_RS04685	0.528	0.000297	gene934	Rubrerythrin	WP_012419913.1
AMUC_RS10850	0.527	1.96E-08	gene2163	Pyruvate carboxyltransferase	WP_012421058.1
AMUC_RS10115	0.524	0.001242	gene2018	Hypothetical protein	WP_042448335.1
AMUC_RS11960	0.519	4.89E-08	gene1079	Hypothetical protein	WP_052294452.1
AMUC_RS00080	0.519	7.96E-05	gene15	DEAD/DEAH box helicase	WP_012419077.1
AMUC_RS11150	0.517	1.37E-15	gene2224	Group 1 glycosyl transferase	WP_012421112.1
AMUC_RS00100	0.514	0.003123	gene19	Hypothetical protein	WP_012419080.1
AMUC_RS09520	0.514	1.88E-07	gene1896	Hypothetical protein	WP_012420817.1
AMUC_RS09040	0.511	7.73E-15	gene1800	2-oxoglutarate dehydrogenase subunit E1	WP_012420726.1
AMUC_RS09485	0.509	4.9E-11	gene1889	VWA domain-containing protein	WP_012420810.1
AMUC_RS04170	0.506	0.006872	gene832	Acyltransferase	WP_012419823.1
AMUC_RS00725	0.506	0.005835	gene145	G-D-S-L family lipolytic protein	WP_012419184.1
AMUC_RS05685	0.496	2.96E-06	gene1133	Hypothetical protein	WP_042447882.1
AMUC_RS10895	0.495	1.1E-16	gene2172	Efflux RND transporter permease subunit	WP_012421066.1
AMUC_RS10930	0.493	5.1E-11	gene2179	Chloride channel protein	WP_012421073.1
AMUC_RS11025	0.493	0.00021	gene2198	Membrane protein	WP_012421089.1
AMUC_RS07000	0.492	1.02E-06	gene1392	Galactose mutarotase	WP_012420348.1
AMUC_RS02265	0.486	1.68E-08	gene451	Hypothetical protein	WP_012419463.1
AMUC_RS09190	0.485	1.27E-11	gene1830	Exodeoxyribonuclease VII large subunit	WP_012420752.1
AMUC_RS01925	0.485	2.63E-06	gene383	DNA polymerase III subunit epsilon	WP_012419399.1
AMUC_RS07990	0.482	0.000377	gene1589	Hypothetical protein	WP_012420529.1
AMUC_RS06395	0.477	0.000109	gene1273	Cobalt ABC transporter ATP-binding protein	WP_012420238.1
AMUC_RS11940	0.475	1E-18	gene1019	Biotin/lipoyl attachment domain-containing protein	WP_012419994.1
AMUC_RS05835	0.475	3.83E-06	gene1161	Hypothetical protein	WP_012420127.1
AMUC_RS01610	0.475	1.21E-05	gene319	Hypothetical protein	WP_012419340.1

AMUC_RS10700	0.474	0.00372	gene2134	Integrase	WP_012421030.1
AMUC_RS02620	0.474	9.63E-15	gene522	Hypothetical protein	WP_022198495.1
AMUC_RS08905	0.474	0.007079	gene1773	Beta-N-acetylhexosaminidase	WP_012420702.1
AMUC_RS04570	0.472	0.002325	gene911	Hypothetical protein	WP_042447790.1
AMUC_RS11905	0.471	2.67E-07	gene958	Merr family transcriptional regulator	WP_012419936.1
AMUC_RS09180	0.469	0.003324	gene1828	Hypothetical protein	WP_042448250.1
AMUC_RS06830	0.466	0.000259	gene1358	Hypothetical protein	WP_012420314.1
AMUC_RS02030	0.465	1.44E-08	gene404	Short-chain dehydrogenase/reductase SDR	WP_012419417.1
AMUC_RS02950	0.465	7.76E-08	gene588	Hypothetical protein	WP_052294428.1
AMUC_RS03840	0.464	4.5E-06	gene765	Hypothetical protein	WP_012419758.1
AMUC_RS10260	0.458	4.9E-06	gene2047	Hypothetical protein	WP_012420950.1
AMUC_RS10145	0.458	0.04025	gene2024	Hypothetical protein	WP_012420928.1
AMUC_RS06125	0.456	0.000308	gene1219	Hypothetical protein	WP_012420187.1
AMUC_RS09720	0.455	8.39E-09	gene1936	SAM-dependent methyltransferase	WP_012420853.1
AMUC_RS07040	0.453	3.8E-10	gene1400	Hypothetical protein	WP_042448011.1
AMUC_RS01305	0.449	2.42E-13	gene258	Peptide deformylase	WP_012419290.1
AMUC_RS01400	0.448	0.025297	gene277	Hypothetical protein	WP_012419308.1
AMUC_RS06995	0.447	5.62E-05	gene1391	Hypothetical protein	WP_042448007.1
AMUC_RS03380	0.446	0.048395	gene674	PEP-CTERM domain protein	WP_012419674.1
AMUC_RS08895	0.444	0.030279	gene1771	Glycoside hydrolase family 2	WP_012420700.1
AMUC_RS10935	0.443	0.017016	gene2180	Hypothetical protein	WP_042448409.1
AMUC_RS12145	0.436	0.041772	gene1958	Hypothetical protein	WP_031931303.1
AMUC_RS10165	0.436	6.82E-13	gene2028	Hypothetical protein	WP_042448337.1
AMUC_RS04120	0.434	5.9E-11	gene821	Hypothetical protein	WP_042447749.1
AMUC_RS03360	0.434	0.000918	gene670	Hypothetical protein	WP_012419669.1
AMUC_RS09770	0.432	5.83E-13	gene1946	Class II aldolase/adducin family protein	WP_012420861.1
AMUC_RS03010	0.432	0.006901	gene600	Hypothetical protein	WP_042447636.1
AMUC_RS06825	0.427	0.001518	gene1357	Hypothetical protein	WP_042447999.1
AMUC_RS12210	0.425	1.75E-09	gene2178	PTS transporter subunit IIA-like nitrogen-regulatory protein ptsn	WP_012421072.1
AMUC_RS03345	0.418	4.34E-07	gene667	Hypothetical protein	WP_042447669.1
AMUC_RS02450	0.417	1.48E-09	gene488	Hypothetical protein	WP_051729260.1
AMUC_RS11145	0.417	0.003834	gene2223	Glycosyl transferase family 1	WP_012421111.1
AMUC_RS06905	0.415	0.009792	gene1373	Hypothetical protein	WP_052294463.1
AMUC_RS11430	0.413	6.18E-08	gene2280	Hypothetical protein	WP_012421164.1
AMUC_RS02835	0.412	0.000106	gene565	Hypothetical protein	WP_052294427.1
AMUC_RS04595	0.410	4.21E-08	gene916	Hypothetical protein	WP_012419896.1
AMUC_RS07860	0.410	1.38E-10	gene1563	Hypothetical protein	WP_012420504.1
AMUC_RS12170	0.409	2.55E-12	gene1998	Hypothetical protein	WP_012420904.1
AMUC_RS08165	0.409	6.8E-16	gene1625	Glyceraldehyde 3-phosphate reductase	WP_012420563.1
AMUC_RS08625	0.405	2.16E-09	gene1717	Polysaccharide deacetylase	WP_012420651.1
AMUC_RS07105	0.404	6.77E-07	gene1413	DNA polymerase IV	WP_012420369.1

AMUC_RS10905	0.402	0.003577	gene2174	PEP-CTERM sorting domain-containing protein	WP_012421068.1
AMUC_RS10460	0.402	0.000323	gene2087	PA14 domain-containing protein	WP_012420987.1
AMUC_RS07920	0.401	0.010281	gene1575	Hypothetical protein	WP_012420515.1
AMUC_RS01650	0.401	0.013286	gene327	Hypothetical protein	WP_042447545.1
AMUC_RS10745	0.397	1.22E-10	gene2143	Hydroxyethylthiazole kinase	WP_012421040.1
AMUC_RS10900	0.396	5.46E-11	gene2173	Efflux transporter outer membrane subunit	
AMUC_RS04815	0.393	0.000726	gene960	Multidrug efflux RND transporter permease subunit	WP_012419938.1
AMUC_RS11515	0.393	2.47E-11	gene2297	Hypothetical protein	WP_012421180.1
AMUC_RS11850	0.393	1.89E-09	gene802	Prepilin-type N-terminal cleavage/methylation domain-containing protein	WP_012419796.1
AMUC_RS09115	0.393	0.037731	gene1815	Nucleotide pyrophosphatase	WP_042448241.1
AMUC_RS05305	0.389	0.001941	gene1059	Hypothetical protein	WP_051729438.1
AMUC_RS03300	0.383	0.017407	gene658	Hypothetical protein	WP_042447666.1
AMUC_RS11385	0.383	6.6E-11	gene2271	Hypothetical protein	WP_042448466.1
AMUC_RS09765	0.382	0.01556	gene1945	Hypothetical protein	WP_031931297.1
AMUC_RS03640	0.381	1.43E-09	gene725	Trypsin-like protein serine protease	WP_012419722.1
AMUC_RS04975	0.380	3.96E-06	gene993	Non-specific serine/threonine protein kinase	WP_012419968.1
AMUC_RS02860	0.375	2.22E-09	gene570	UBA/THIF-type NAD/FAD binding protein	WP_012419575.1
AMUC_RS03165	0.373	1.09E-06	gene631	Hypothetical protein	WP_012419629.1
AMUC_RS00805	0.373	0.026264	gene160	Hypothetical protein	WP_012419197.1
AMUC_RS12255	0.371	0.001004	gene2312	Chloramphenicol phosphotransferase	WP_012421196.1
AMUC_RS09050	0.369	4.84E-07	gene1802	Cytochrome d ubiquinol oxidase subunit I	WP_012420728.1
AMUC_RS02840	0.368	0.005247	gene566	Hypothetical protein	WP_012419571.1
AMUC_RS09495	0.367	7.3E-09	gene1891	Hypothetical protein	WP_012420812.1
AMUC_RS07640	0.363	1.2E-08	gene1519	Phosphate acetyltransferase	WP_012420465.1
AMUC_RS10740	0.363	1.16E-06	gene2142	Thiamine phosphate synthase	WP_012421039.1
AMUC_RS04405	0.361	7.13E-06	gene878	Hypothetical protein	WP_012419865.1
AMUC_RS11915	0.359	1.21E-05	gene968	Hypothetical protein	WP_052294446.1
AMUC_RS00610	0.358	1.97E-05	gene122	Hypothetical protein	WP_012419164.1
AMUC_RS04980	0.354	6.42E-07	gene994	Bifunctional metallophosphatase/5'-nucleotidase	WP_012419969.1
AMUC_RS09065	0.354	0.000176	gene1805	Hypothetical protein	WP_012420731.1
AMUC_RS07730	0.351	1.47E-12	gene1537	Hypothetical protein	WP_012420480.1
AMUC_RS08860	0.348	1.8E-06	gene1764	Hypothetical protein	WP_042448209.1
AMUC_RS11505	0.348	3.24E-11	gene2295	NADH-quinone oxidoreductase subunit B	WP_012421178.1
AMUC_RS10270	0.346	0.001495	gene2049	Hypothetical protein	WP_012420952.1
AMUC_RS06340	0.345	2.03E-06	gene1262	Hypothetical protein	WP_012420228.1
AMUC_RS06810	0.343	0.005558	gene1354	Hypothetical protein	WP_012420310.1
AMUC_RS04115	0.342	0.006873	gene820	Hypothetical protein	WP_012419814.1

AMUC_RS03020	0.341	1.41E-08	gene602	Phage SPO1 DNA polymerase-related protein	WP_012419604.1
AMUC_RS11720	0.336	8.42E-06	gene228	Biotin--[acetyl-coa-carboxylase] ligase	WP_012419264.1
AMUC_RS08500	0.336	3.51E-08	gene1692	Lipid II flippase murj	WP_012420625.1
AMUC_RS07655	0.334	0.000198	gene1522	Hypothetical protein	WP_012420468.1
AMUC_RS03365	0.333	0.003405	gene671	Type 11 methyltransferase	WP_012419670.1
AMUC_RS02445	0.333	2.27E-06	gene487	DNA protecting protein dpra	WP_012419496.1
AMUC_RS08610	0.332	0.00198	gene1714	NADH dehydrogenase (ubiquinone)	WP_012420648.1
AMUC_RS07785	0.332	2.65E-12	gene1548	Excinuclease ABC subunit A	WP_012420489.1
AMUC_RS09295	0.330	2.54E-05	gene1851	Hypothetical protein	WP_012420772.1
AMUC_RS04165	0.330	3.83E-05	gene831	2-hydroxyacid dehydrogenase	WP_031930511.1
AMUC_RS09705	0.328	0.000269	gene1933	Hypothetical protein	WP_042448315.1
AMUC_RS06505	0.328	0.007528	gene1294	Hypothetical protein	WP_012420259.1
AMUC_RS05320	0.328	0.000436	gene1062	PEP-CTERM domain protein	WP_012420034.1
AMUC_RS00085	0.328	1.65E-06	gene16	Hypothetical protein	WP_012419078.1
AMUC_RS11310	0.327	5.43E-09	gene2256	ATPase AAA	WP_012421142.1
AMUC_RS09575	0.327	0.000425	gene1907	Hypothetical protein	WP_042448293.1
AMUC_RS02135	0.325	0.008998	gene425	30S ribosomal protein S15	WP_012419437.1
AMUC_RS09045	0.325	1.88E-07	gene1801	Cytochrome D oxidase subunit I	WP_012420727.1
AMUC_RS10455	0.322	0.004392	gene2086	Hypothetical protein	WP_012420986.1
AMUC_RS11445	0.322	0.011436	gene2283	Hypothetical protein	WP_012421167.1
AMUC_RS06400	0.321	0.006716	gene1274	Cobalt ECF transporter T component cbiq	WP_012420239.1
AMUC_RS11570	0.320	0.001543	gene2308	Hypothetical protein	WP_012421192.1
AMUC_RS11315	0.320	1.68E-09	gene2257	Hypothetical protein	WP_012421143.1
AMUC_RS09795	0.318	3.36E-06	gene1951	Hypothetical protein	WP_012420866.1
AMUC_RS11875	0.318	0.000863	gene863	Hypothetical protein	WP_052294439.1
AMUC_RS06215	0.316	1.31E-05	gene1237	VWA domain-containing protein	WP_012420204.1
AMUC_RS11860	0.315	6.62E-05	gene814	Family 2 glycosyl transferase	WP_012419808.1
AMUC_RS00485	0.313	0.010512	gene97	Hypothetical protein	WP_012419141.1
AMUC_RS01110	0.313	7.06E-09	gene219	Serine/threonine protein kinase	WP_012419255.1
AMUC_RS09230	0.313	1.48E-06	gene1838	Two-component sensor histidine kinase	WP_012420759.1
AMUC_RS10645	0.312	2.79E-08	gene2123	Tryptophanase	WP_012421020.1
AMUC_RS00945	0.311	0.000884	gene187	Hypothetical protein	WP_012419223.1
AMUC_RS11450	0.311	4.03E-05	gene2284	6-pyruvoyltetrahydropterin synthase	WP_012421168.1
AMUC_RS08395	0.310	0.002376	gene1671	Hypothetical protein	WP_042448177.1
AMUC_RS01765	0.307	4.24E-05	gene350	Signal transduction histidine kinase	WP_012419368.1
AMUC_RS03960	0.306	1.78E-05	gene789	Hypothetical protein	WP_012419782.1
AMUC_RS09855	0.304	3.84E-11	gene1964	PDZ/DHR/GLGF domain-containing protein	WP_012420875.1
AMUC_RS11810	0.303	0.000215	gene614	Hypothetical protein	WP_052294431.1
AMUC_RS05715	0.303	6E-06	gene1139	Transcriptional regulator	WP_012420108.1
AMUC_RS01070	0.302	0.012885	gene211	Peptidase M28	WP_012419246.1
AMUC_RS03500	0.301	0.00154	gene697	tRNA dimethylallyl transferase	WP_012419694.1

AMUC_RS00960	0.300	1.11E-05	gene190	Peptidyl-prolyl cis-trans isomerase	WP_012419226.1
AMUC_RS08115	0.300	2.7E-05	gene1614	Signal transduction histidine kinase	WP_012420552.1
AMUC_RS08375	0.299	3.46E-08	gene1667	Alpha-2-macroglobulin domain-containing protein	WP_012420601.1
AMUC_RS01410	0.298	0.00585	gene279	Hypothetical protein	WP_012419310.1
AMUC_RS06265	0.298	0.024896	gene1247	Hypothetical protein	WP_012420214.1
AMUC_RS08980	0.298	0.001	gene1788	Cobalamin biosynthesis protein CobD	WP_012420715.1
AMUC_RS02250	0.297	6.95E-05	gene448	ABC transporter permease	WP_012419460.1
AMUC_RS11560	0.296	2.03E-05	gene2306	Hypothetical protein	WP_012421189.1
AMUC_RS09685	0.295	0.007949	gene1929	Hypothetical protein	WP_042448310.1
AMUC_RS08520	0.295	1.19E-05	gene1696	NCAIR mutase-like protein	WP_012420629.1
AMUC_RS02435	0.293	0.000299	gene485	Hypothetical protein	WP_051729257.1
AMUC_RS11800	0.293	7.82E-06	gene599	Hypothetical protein	WP_031930332.1
AMUC_RS06175	0.292	0.00979	gene1229	Hypothetical protein	WP_012420197.1
AMUC_RS03805	0.291	3.7E-06	gene758	Hypothetical protein	WP_052294496.1
AMUC_RS02625	0.291	2.68E-09	gene523	Hypothetical protein	WP_012419531.1
AMUC_RS10195	0.288	1.41E-07	gene2034	Hypothetical protein	WP_012420938.1
AMUC_RS03255	0.287	1.59E-05	gene649	DNA recombination protein recf	WP_012419648.1
AMUC_RS07100	0.287	0.001598	gene1412	Methionine sulfoxide reductase B	WP_012420368.1
AMUC_RS06220	0.287	0.002375	gene1238	Hypothetical protein	WP_012420205.1
AMUC_RS03305	0.286	0.048749	gene659	Hypothetical protein	WP_052294433.1
AMUC_RS12130	0.282	0.003827	gene1879	Hypothetical protein	WP_012420800.1
AMUC_RS07410	0.282	0.031557	gene1473	Hypothetical protein	WP_012420430.1
AMUC_RS03600	0.280	0.000112	gene717	Polypeptide-transport-associated domain-containing protein FtsQ-type	WP_012419714.1
AMUC_RS00690	0.280	1.02E-05	gene138	Hypothetical protein	WP_012419179.1
AMUC_RS06115	0.279	0.001139	gene1217	Hypothetical protein	WP_012420186.1
AMUC_RS08355	0.278	1.16E-05	gene1663	ATP-dependent nuclease subunit B-like protein	WP_012420598.1
AMUC_RS04985	0.277	0.002721	gene995	Hypothetical protein	WP_051729401.1
AMUC_RS02650	0.277	0.001542	gene528	Alpha/beta superfamily hydrolase	WP_012419535.1
AMUC_RS00185	0.276	0.000215	gene36	Proline--tRNA ligase	WP_012419096.1
AMUC_RS03175	0.276	0.003548	gene633	Hypothetical protein	WP_042447658.1
AMUC_RS12075	0.276	0.013249	gene1579	Hypothetical protein	WP_012420519.1
AMUC_RS03580	0.275	3.1E-07	gene713	Cell cycle protein	WP_012419710.1
AMUC_RS07790	0.272	0.006316	gene1549	Hypothetical protein	WP_042448097.1
AMUC_RS11040	0.271	3.04E-05	gene2201	Hypothetical protein	WP_012421091.1
AMUC_RS00695	0.270	1.49E-05	gene139	AI-2E family transporter	WP_012419180.1
AMUC_RS06010	0.270	2.1E-08	gene1196	Terpene cyclase/mutase family protein	WP_012420164.1
AMUC_RS08160	0.269	2.14E-07	gene1624	Lipoate-protein ligase A-like protein	WP_012420562.1
AMUC_RS00325	0.268	0.000209	gene65	Zinc transporter	WP_012419116.1
AMUC_RS00955	0.268	0.002805	gene189	Twitching motility protein PilT	WP_012419225.1
AMUC_RS11365	0.268	6.6E-06	gene2267	Hydrogenase 2 large subunit	WP_012421152.1

AMUC_RS03880	0.268	6.56E-08	gene773	Hypothetical protein	WP_012419766.1
AMUC_RS04840	0.267	3.34E-05	gene965	Membrane protein	WP_012419942.1
AMUC_RS09500	0.267	2.68E-06	gene1892	ATPase AAA	WP_012420813.1
AMUC_RS02970	0.266	0.003637	gene592	Hypothetical protein	WP_012419596.1
AMUC_RS11660	0.266	0.002513	gene115	Hypothetical protein	WP_052294408.1
AMUC_RS04900	0.265	0.027613	gene977	Hypothetical protein	WP_012419954.1
AMUC_RS06495	0.263	1.1E-05	gene1292	Hypothetical protein	WP_042447972.1
AMUC_RS10265	0.262	3.06E-05	gene2048	Beta-N-acetylhexosaminidase	WP_012420951.1
AMUC_RS01225	0.262	0.00098	gene242	Acriflavin resistance protein	WP_012419278.1
AMUC_RS01895	0.262	0.00021	gene377	Phospholipid/glycerol acyltransferase	WP_012419393.1
AMUC_RS06405	0.262	8.44E-06	gene1275	Cobalamin biosynthesis protein CbiM	WP_012420240.1
AMUC_RS10040	0.262	0.012753	gene2002	Hypothetical protein	WP_012420909.1
AMUC_RS08275	0.261	0.020066	gene1647	Hypothetical protein	WP_042448157.1
AMUC_RS01150	0.261	0.018565	gene227	Glutaconyl-coa decarboxylase subunit beta	
AMUC_RS06015	0.260	6.75E-08	gene1197	Terpene cyclase/mutase family protein	WP_012420165.1
AMUC_RS01505	0.260	0.003954	gene298	rRNA maturation RNase YbeY	WP_052294414.1
AMUC_RS00520	0.260	0.001754	gene104	DNA mismatch repair protein MutL	WP_012419146.1
AMUC_RS11360	0.260	2.42E-06	gene2266	Ni/Fe hydrogenase	WP_012421151.1
AMUC_RS05650	0.260	9.58E-07	gene1126	Methylenetetrahydrofolate reductase [NAD(P)H]	WP_012420096.1
AMUC_RS00950	0.260	5.17E-07	gene188	Twitching motility protein PilT	WP_012419224.1
AMUC_RS03340	0.259	0.01087	gene666	DNA-deoxyinosine glycosylase	WP_012419665.1
AMUC_RS08745	0.258	1.7E-06	gene1741	Hypothetical protein	WP_052294476.1
AMUC_RS05120	0.258	0.000128	gene1022	Hypothetical protein	WP_012419997.1
AMUC_RS00790	0.258	4.7E-07	gene157	Hypothetical protein	WP_012419194.1
AMUC_RS07915	0.257	8.6E-05	gene1574	4-hydroxythreonine-4-phosphate dehydrogenase PdxA	WP_012420514.1
AMUC_RS05280	0.255	1.46E-05	gene1054	Sugar-binding protein	WP_012420027.1
AMUC_RS09370	0.255	8.8E-06	gene1866	Sulfatase	WP_012420787.1
AMUC_RS01335	0.255	1.05E-05	gene264	MBL fold hydrolase	WP_012419296.1
AMUC_RS06045	0.255	0.006106	gene1203	MATE family efflux transporter	WP_012420172.1
AMUC_RS11355	0.252	0.015782	gene2265	Carbohydrate porin	WP_012421150.1
AMUC_RS11935	0.252	0.001924	gene1017	Family 2 glycosyl transferase	WP_012419992.1
AMUC_RS01220	0.252	0.000584	gene241	RND family efflux transporter MFP subunit	WP_012419277.1
AMUC_RS04780	0.251	0.000137	gene953	Hypothetical protein	WP_012419932.1
AMUC_RS08440	0.250	0.017267	gene1680	Pseudouridine synthase	WP_012420613.1
AMUC_RS08495	0.250	0.000127	gene1691	Aspartate aminotransferase	WP_012420624.1
AMUC_RS00970	0.247	0.004085	gene192	Hypothetical protein	WP_012419227.1
AMUC_RS00905	0.247	0.000334	gene179	ABC transporter	WP_012419215.1
AMUC_RS08460	0.245	0.000201	gene1684	Glycosyl transferase family 2	WP_012420617.1
AMUC_RS09275	0.243	0.004536	gene1847	Hypothetical protein	WP_012420768.1

AMUC_RS08350	0.242	1.02E-05	gene1662	Uvrd/REP helicase	WP_012420597.1
AMUC_RS05975	0.242	0.000686	gene1189	Uracil-DNA glycosylase	WP_012420157.1
AMUC_RS11740	0.240	0.005764	gene368	Hypothetical protein	WP_012419385.1
AMUC_RS12015	0.240	2.63E-06	gene1325	N-acetylmuramoyl-L-alanine amidase	WP_012420284.1
AMUC_RS10565	0.239	5.93E-06	gene2107	Hypothetical protein	WP_012421004.1
AMUC_RS03610	0.238	0.000537	gene719	Cell wall hydrolase/autolysin	WP_012419716.1
AMUC_RS00615	0.237	0.002286	gene123	RND transporter NodT	WP_012419165.1
AMUC_RS08035	0.237	0.001791	gene1598	Polysaccharide deacetylase	WP_012420537.1
AMUC_RS09925	0.237	0.011485	gene1979	Hypothetical protein	WP_012420885.1
AMUC_RS08020	0.236	5.73E-05	gene1595	Hypothetical protein	WP_012420534.1
AMUC_RS10845	0.235	0.004656	gene2162	Cardiolipin synthase	WP_052294493.1
AMUC_RS01210	0.235	0.003483	gene239	Hypothetical protein	WP_012419275.1
AMUC_RS03800	0.235	0.003773	gene757	Hypothetical protein	WP_012419752.1
AMUC_RS06210	0.233	0.013164	gene1236	Hypothetical protein	WP_042447945.1
AMUC_RS02995	0.232	1.36E-07	gene597	MotA/TolQ/ExbB proton channel	WP_012419601.1
AMUC_RS11780	0.231	0.000193	gene468	Transcription antitermination factor NusB	WP_012419477.1
AMUC_RS07875	0.231	0.010441	gene1566	Exosortase	WP_042448118.1
AMUC_RS09185	0.229	0.002142	gene1829	Hypothetical protein	WP_042448252.1
AMUC_RS05170	0.228	0.000226	gene1032	Tat (twin-arginine translocation) pathway signal sequence	WP_042448853.1
AMUC_RS11215	0.228	0.000106	gene2237	Cystathionine beta-lyase	WP_012421125.1
AMUC_RS03965	0.228	0.026884	gene790	Hypothetical protein	WP_042447734.1
AMUC_RS06245	0.226	0.00111	gene1243	ATPase AAA	WP_042448944.1
AMUC_RS10385	0.226	0.000197	gene2072	Hypothetical protein	WP_012420972.1
AMUC_RS08515	0.226	0.000129	gene1695	Hypothetical protein	WP_012420628.1
AMUC_RS08095	0.224	0.002023	gene1610	OmpA/MotB domain-containing protein	WP_012420548.1
AMUC_RS11680	0.223	1.2E-08	gene146	Hypothetical protein	WP_052294412.1
AMUC_RS03755	0.223	0.002399	gene748	Thiol reductase thioredoxin	WP_012419743.1
AMUC_RS08400	0.222	0.002553	gene1672	Hypothetical protein	WP_012420605.1
AMUC_RS11165	0.221	0.038533	gene2227	Transferase	WP_012421115.1
AMUC_RS04990	0.221	6.67E-05	gene996	ATP-NAD/acox kinase	WP_012419971.1
AMUC_RS11995	0.220	4.76E-05	gene1195	Hypothetical protein	WP_052294455.1
AMUC_RS08000	0.217	0.008986	gene1591	1,4-dihydroxy-2-naphthoate octaprenyltransferase	WP_012420531.1
AMUC_RS03210	0.216	0.001073	gene640	Maf-like protein	WP_012419638.1
AMUC_RS10595	0.216	0.002987	gene2113	Hypothetical protein	WP_042448388.1
AMUC_RS02035	0.216	0.001754	gene405	Hypothetical protein	WP_012419418.1
AMUC_RS09220	0.215	0.004583	gene1836	Acyl-ACP thioesterase	WP_012420757.1
AMUC_RS07620	0.215	0.000805	gene1515	Hypothetical protein	WP_052294501.1
AMUC_RS04245	0.214	0.025088	gene847	Rhomboid family intramembrane serine protease	WP_012419836.1

AMUC_RS12250	0.214	0.000212	gene2299	Acyl-Coa synthetase (AMP-forming)/AMP-acid ligase II-like protein	WP_012421182.1
AMUC_RS09515	0.213	0.003271	gene1895	Hypothetical protein	WP_012420816.1
AMUC_RS09235	0.211	0.002065	gene1839	DNA-binding response regulator	WP_012420760.1
AMUC_RS08480	0.211	6.73E-06	gene1688	Type II secretion system protein E	WP_012420621.1
AMUC_RS12060	0.211	0.046325	gene1512	Hypothetical protein	WP_052294466.1
AMUC_RS03775	0.211	0.004392	gene752	Terpene cyclase/mutase family protein	WP_012419747.1
AMUC_RS07850	0.211	5.3E-05	gene1561	Signal peptidase I	WP_042448113.1
AMUC_RS11405	0.211	9.08E-05	gene2275	NAD(P)H-dependent oxidoreductase	WP_012421160.1
AMUC_RS11375	0.210	0.000519	gene2269	Hydrogenase maturation protease	WP_012421154.1
AMUC_RS12080	0.209	0.001299	gene1601	Peptidase A24A domain-containing protein	WP_012420539.1
AMUC_RS09010	0.209	4.01E-05	gene1794	Hypothetical protein	WP_012420720.1
AMUC_RS09415	0.208	0.002855	gene1875	Hypothetical protein	WP_012420796.1
AMUC_RS02050	0.207	0.001636	gene408	Chloride channel protein	WP_012419421.1
AMUC_RS05465	0.207	0.005685	gene1089	Hypothetical protein	WP_042447864.1
AMUC_RS03315	0.206	0.009353	gene661	Rrna (cytidine-2'-O-)-methyltransferase	WP_012419660.1
AMUC_RS07855	0.205	0.001056	gene1562	Glycolate oxidase	WP_012420503.1
AMUC_RS06365	0.205	0.001395	gene1267	Mannose-1-phosphate guanyltransferase	WP_012420232.1
AMUC_RS06320	0.204	0.00034	gene1258	Phosphopyruvate hydratase	WP_042448954.1
AMUC_RS09620	0.203	0.036821	gene1916	Metallophosphoesterase	WP_012420835.1
AMUC_RS10350	0.203	0.001419	gene2065	Hypothetical protein	WP_042448356.1
AMUC_RS04750	0.202	0.006601	gene947	Hypothetical protein	WP_012419926.1
AMUC_RS01205	0.202	0.019451	gene238	Hypothetical protein	WP_012419274.1
AMUC_RS05230	0.202	0.000974	gene1044	Monogalactosyldiacylglycerol synthase	WP_012420020.1
AMUC_RS05905	0.202	0.006328	gene1175	Cell division protein FtsA	WP_012420142.1
AMUC_RS00600	0.202	0.008758	gene120	RND family efflux transporter MFP subunit	WP_012419162.1
AMUC_RS04785	0.201	0.000334	gene954	Catalytic domain of components of various dehydrogenase complexes	WP_012419933.1
AMUC_RS00240	0.201	0.004851	gene47	Peptide chain release factor 1	WP_012419103.1
AMUC_RS05175	0.200	0.003926	gene1033	Hypothetical protein	WP_052294451.1
AMUC_RS04895	0.200	0.000123	gene976	Peptide methionine sulfoxide reductase MsrB	WP_012419953.1
AMUC_RS00040	0.200	0.00042	gene7	Hypothetical protein	WP_012419068.1
AMUC_RS00120	0.200	0.038385	gene23	G-D-S-L family lipolytic protein	WP_012419084.1
AMUC_RS03235	0.197	0.002528	gene645	Thiamine-monophosphate kinase	WP_012419644.1
AMUC_RS00045	0.197	0.00615	gene8	Hypothetical protein	WP_012419069.1
AMUC_RS10095	0.197	0.001455	gene2013	Hypothetical protein	WP_012420919.1
AMUC_RS09250	0.196	0.013931	gene1842	Hypothetical protein	WP_012420763.1
AMUC_RS08760	0.196	0.001599	gene1744	Hypothetical protein	WP_012420675.1
AMUC_RS03155	0.195	0.004933	gene629	Tetraacyldisaccharide 4'-kinase	WP_012419627.1
AMUC_RS03550	0.195	0.000338	gene707	Peptidoglycan glycosyltransferase	WP_012419704.1

AMUC_RS03615	0.195	0.004957	gene720	Lipoyl(octanoyl) transferase	WP_012419717.1
AMUC_RS08755	0.194	0.000265	gene1743	Hypothetical protein	WP_042448202.1
AMUC_RS07720	0.194	0.015472	gene1535	RNA pseudouridine synthase	WP_012420478.1
AMUC_RS02815	0.194	0.001004	gene561	Peptidoglycan glycosyltransferase	WP_012419567.1
AMUC_RS11970	0.194	0.023512	gene1150	Group 1 glycosyl transferase	WP_012420118.1
AMUC_RS03845	0.194	0.031194	gene766	Radical SAM protein	WP_012419759.1
AMUC_RS00095	0.193	1.92E-05	gene18	Glycosyl hydrolase family 109 protein 1	WP_012419079.1
AMUC_RS10975	0.193	0.033873	gene2188	Rubrerythrin	WP_042448418.1
AMUC_RS00785	0.193	0.001495	gene156	Hypothetical protein	WP_042447469.1
AMUC_RS00710	0.193	0.014881	gene142	Hypothetical protein	WP_052294411.1
AMUC_RS09475	0.191	0.000409	gene1887	Hypothetical protein	WP_012420808.1
AMUC_RS07050	0.191	0.044265	gene1402	Ribosome recycling factor	WP_012420357.1
AMUC_RS01020	0.191	0.049097	gene202	Hypothetical protein	WP_012419237.1
AMUC_RS00330	0.191	0.034538	gene66	Manganese ABC transporter permease	WP_012419117.1
AMUC_RS04935	0.189	0.009642	gene984	Cupin	WP_012419961.1
AMUC_RS10395	0.188	0.030814	gene2074	N-acylglucosamine 2-epimerase	WP_012420974.1
AMUC_RS08985	0.187	0.001628	gene1789	Cobyric acid synthase	WP_012420716.1
AMUC_RS00940	0.187	0.032378	gene186	Hypothetical protein	WP_012419222.1
AMUC_RS11320	0.186	0.004022	gene2258	Peptidyl-tRNA hydrolase	WP_012421144.1
AMUC_RS11370	0.185	0.001338	gene2268	Hypothetical protein	WP_031931547.1
AMUC_RS03585	0.184	0.00385	gene714	UDP-N-acetylglucosamine--N-acetylmuramyl-(pentapeptide) pyrophosphoryl-undecaprenol N-acetylglucosamine transferase	WP_012419711.1
AMUC_RS11395	0.183	0.036992	gene2273	Hypothetical protein	WP_012421158.1
AMUC_RS02765	0.182	0.001449	gene551	DUF5069 domain-containing protein	WP_012419557.1
AMUC_RS09245	0.182	0.000839	gene1841	Delta-aminolevulinic acid dehydratase	WP_031931218.1
AMUC_RS09035	0.181	0.009116	gene1799	Dihydrolipoamide succinyltransferase	WP_012420725.1
AMUC_RS04095	0.180	0.021449	gene816	Hypothetical protein	WP_042447747.1
AMUC_RS01990	0.179	0.001012	gene396	Hypothetical protein	WP_012419410.1
AMUC_RS08655	0.177	0.004709	gene1723	4-alpha-glucanotransferase	WP_012420656.1
AMUC_RS11155	0.177	0.049503	gene2225	Group 1 glycosyl transferase	WP_012421113.1
AMUC_RS01900	0.177	0.019362	gene378	Deoxycytidylate deaminase	WP_012419394.1
AMUC_RS02560	0.177	0.044701	gene510	Peptidase M23	WP_012419518.1
AMUC_RS09800	0.177	0.004696	gene1952	Exo-alpha-sialidase	WP_012420867.1
AMUC_RS10405	0.176	0.015804	gene2076	Aminodeoxychorismate synthase component I	WP_012420976.1
AMUC_RS02850	0.176	0.003867	gene568	Hypothetical protein	WP_012419573.1
AMUC_RS09280	0.176	0.007349	gene1848	1-deoxy-D-xylulose-5-phosphate reductoisomerase	WP_012420769.1
AMUC_RS03265	0.175	0.027309	gene651	Ribonuclease III	WP_052294432.1
AMUC_RS07610	0.174	0.001328	gene1513	Hypothetical protein	WP_052294467.1
AMUC_RS07245	0.173	0.034492	gene1441	Hypothetical protein	WP_012420399.1

AMUC_RS11715	0.172	0.001428	gene218	Hypothetical protein	WP_012419253.1
AMUC_RS10605	0.171	0.018065	gene2115	Terpene cyclase/mutase family protein	WP_012421012.1
AMUC_RS08535	0.170	0.000383	gene1699	Hypothetical protein	WP_052294473.1
AMUC_RS07085	0.169	0.005973	gene1409	Recombination protein RecR	WP_012420364.1
AMUC_RS04710	0.167	0.007818	gene939	Alkaline phosphatase	WP_012419918.1
AMUC_RS01585	0.167	0.00426	gene314	DNA mismatch repair protein mutt	WP_012419335.1
AMUC_RS10765	0.166	0.009634	gene2147	Beta-N-acetylhexosaminidase	WP_012421043.1
AMUC_RS02790	0.166	0.024533	gene556	F0F1-type ATP synthase delta subunit-like protein	WP_012419562.1
AMUC_RS09440	0.165	0.020644	gene1880	Serine/threonine protein kinase	WP_012420801.1
AMUC_RS11285	0.164	0.014789	gene2251	Hypothetical protein	WP_012421137.1
AMUC_RS08775	0.164	0.01087	gene1747	Diacylglycerol kinase catalytic subunit	WP_012420678.1
AMUC_RS05125	0.164	0.024896	gene1023	Carbon starvation protein csta	WP_012419998.1
AMUC_RS06795	0.164	0.010932	gene1351	Hypothetical protein	WP_012420307.1
AMUC_RS11280	0.160	0.017098	gene2250	Coproporphyrinogen III oxidase	WP_012421136.1
AMUC_RS05695	0.160	0.049012	gene1135	MFS transporter	WP_012420105.1
AMUC_RS01185	0.159	0.029775	gene234	Single-stranded DNA-binding protein	WP_012419270.1
AMUC_RS10780	0.159	0.005298	gene2150	Hypothetical protein	WP_042448395.1
AMUC_RS03085	0.159	0.006033	gene616	Prepilin-type N-terminal cleavage/methylation domain-containing protein	WP_012419615.1
AMUC_RS03025	0.158	0.033808	gene603	Indole-3-glycerol-phosphate synthase	WP_012419605.1
AMUC_RS10415	0.158	0.001581	gene2078	12-oxophytodienoate reductase	WP_042449258.1
AMUC_RS03570	0.158	0.005061	gene711	UDP-N-acetylmuramoylalanine--D-glutamate ligase	WP_012419708.1
AMUC_RS04480	0.155	0.002142	gene893	Potassium-transporting atpase C subunit	WP_012419880.1
AMUC_RS09505	0.154	0.013092	gene1893	Glutamyl-tRNA(Gln) amidotransferase subunit A	WP_012420814.1
AMUC_RS07960	0.154	0.046152	gene1583	Hypothetical protein	WP_012420523.1
AMUC_RS03760	0.151	0.003271	gene749	Hypothetical protein	WP_031930442.1
AMUC_RS05490	0.150	0.042967	gene1094	Hypothetical protein	WP_042447867.1
AMUC_RS08005	0.150	0.022459	gene1592	3-dehydroquinase	WP_012420532.1
AMUC_RS01665	0.149	0.046984	gene330	Hypothetical protein	WP_012419349.1
AMUC_RS06760	0.148	0.017819	gene1344	D-hexose-6-phosphate mutarotase	WP_012420301.1
AMUC_RS00620	0.148	0.004311	gene124	EstA family serine hydrolase	WP_012419166.1
AMUC_RS12135	0.147	0.004022	gene1888	VWA domain-containing protein	WP_012420809.1
AMUC_RS08530	0.146	0.048749	gene1698	TIGR00299 family protein	WP_052294472.1
AMUC_RS05910	0.145	0.010228	gene1176	Hypothetical protein	WP_012420143.1
AMUC_RS02440	0.145	0.014482	gene486	Hypothetical protein	WP_052294424.1
AMUC_RS12085	0.145	0.015299	gene1621	Prepilin-type N-terminal cleavage/methylation domain-containing protein	WP_012420559.1

AMUC_RS08575	0.145	0.009569	gene1707	Proton-translocating NADH-quinone oxidoreductase subunit L	WP_012420641.1
AMUC_RS04745	0.144	0.030692	gene946	DNA repair protein RecN	WP_012419925.1
AMUC_RS03535	0.143	0.013947	gene704	MraZ protein	WP_012419701.1
AMUC_RS08865	0.143	0.010643	gene1765	Hypothetical protein	WP_012420694.1
AMUC_RS02190	0.143	0.022721	gene436	Prepilin-type N-terminal cleavage/methylation domain-containing protein	WP_012419448.1
AMUC_RS00065	0.142	0.004741	gene12	DNA ligase (NAD(+)) LigA	WP_012419073.1
AMUC_RS07615	0.142	0.011411	gene1514	Hypothetical protein	WP_012420460.1
AMUC_RS10375	0.141	0.00205	gene2070	Hypothetical protein	WP_012420970.1
AMUC_RS03645	0.140	0.046754	gene726	3-dehydroquinate synthase	WP_012419723.1
AMUC_RS03715	0.137	0.02625	gene740	DNA helicase UvrD	WP_012419735.1
AMUC_RS01930	0.136	0.004592	gene384	DNA mismatch repair protein MutS	WP_012419400.1
AMUC_RS01805	0.135	0.038385	gene358	GTPase Der	WP_012419376.1
AMUC_RS06370	0.134	0.02625	gene1268	Di-trans%2Cpoly-cis-decaprenylcistransferase	WP_012420233.1
AMUC_RS09540	0.133	0.011153	gene1900	Hypothetical protein	WP_012420820.1
AMUC_RS04630	0.131	0.016355	gene923	Glycoside hydrolase	WP_012419903.1
AMUC_RS04500	0.130	0.036482	gene897	ATP-dependent chaperone ClpB	WP_012419884.1
AMUC_RS11955	0.130	0.017057	gene1041	SSS sodium solute transporter superfamily protein	WP_012420017.1
AMUC_RS05995	0.129	0.006316	gene1193	Hypothetical protein	WP_052294454.1
AMUC_RS10660	0.127	0.01976	gene2126	Phenylalanine--tRNA ligase subunit beta	WP_012421023.1
AMUC_RS09990	0.126	0.042311	gene1992	Alpha-glucosidase	WP_012420898.1
AMUC_RS08925	0.124	0.024201	gene1777	6-phosphofructokinase	WP_012420704.1
AMUC_RS09985	0.121	0.003247	gene1991	Group 1 glycosyl transferase	WP_012420897.1
AMUC_RS02485	0.121	0.045917	gene495	Sulfatase	WP_012419504.1
AMUC_RS10510	0.117	0.037262	gene2096	Hypothetical protein	WP_042448368.1
AMUC_RS09405	0.116	0.036195	gene1873	Miro domain-containing protein	WP_012420794.1
AMUC_RS08170	0.114	0.043033	gene1626	Sodium-translocating pyrophosphatase	WP_042449078.1
AMUC_RS09980	0.111	0.020882	gene1990	Glycoside hydrolase	WP_012420896.1
AMUC_RS04790	0.111	0.044701	gene955	Transketolase	WP_012419934.1
AMUC_RS00300	0.110	0.044701	gene60	Single-stranded-DNA-specific exonuclease RecJ	WP_012419112.1
AMUC_RS09090	0.105	0.017697	gene1810	Hypothetical protein	WP_012420737.1
AMUC_RS08750	0.105	0.014075	gene1742	Type II and III secretion system protein	WP_012420673.1
AMUC_RS05510	0.104	0.037398	gene1098	PEP-CTERM domain protein	WP_042447869.1
AMUC_RS02865	0.098	0.047706	gene571	Arabinose-5-phosphate isomerase	WP_012419576.1
**Downregulated genes**					
AMUC_RS02520	-0.099	0.038783	gene502	Phosphoribosylaminoimidazole synthetase	WP_012419510.1
AMUC_RS09350	-0.108	0.03357	gene1862	1%2C4-alpha-glucan-branching protein	WP_012420783.1

AMUC_RS11605	-0.112	0.006846	gene2315	Preprotein translocase subunit SecA	WP_012421198.1
AMUC_RS10640	-0.112	0.048749	gene2122	Serine/threonine protein kinase	WP_012421019.1
AMUC_RS09015	-0.115	0.038496	gene1795	Amidohydrolase	WP_012420721.1
AMUC_RS06585	-0.115	0.007716	gene1310	Hypothetical protein	WP_031930862.1
AMUC_RS09095	-0.116	0.048993	gene1811	Endoribonuclease	WP_042449145.1
AMUC_RS06645	-0.118	0.018065	gene1321	Hypothetical protein	WP_012420280.1
AMUC_RS00745	-0.119	0.011153	gene148	DNA topoisomerase	
AMUC_RS03620	-0.121	0.006504	gene721	3-isopropylmalate dehydratase large subunit	WP_012419718.1
AMUC_RS09425	-0.121	0.014678	gene1877	Glycosyl transferase family 2	WP_012420798.1
AMUC_RS08545	-0.122	0.013263	gene1701	Hypothetical protein	WP_042448189.1
AMUC_RS03690	-0.122	0.046944	gene735	Hypothetical protein	WP_012419730.1
AMUC_RS01005	-0.123	0.024637	gene199	PDZ/DHR/GLGF domain-containing protein	WP_012419234.1
AMUC_RS08810	-0.124	0.004045	gene1754	Acetolactate synthase catalytic subunit	
AMUC_RS00675	-0.128	0.022111	gene135	FAD-binding protein	WP_042448535.1
AMUC_RS03925	-0.128	0.006873	gene782	Branched chain amino acid aminotransferase	WP_012419775.1
AMUC_RS00255	-0.129	0.044265	gene50	Diaminopimelate epimerase	WP_012419106.1
AMUC_RS04380	-0.131	0.035301	gene873	tRNA uridine(34) 5-carboxymethylaminomethyl synthesis enzyme MnmG	WP_012419860.1
AMUC_RS05755	-0.134	0.007966	gene1147	Sulfatase	WP_012420115.1
AMUC_RS12205	-0.136	0.005298	gene2154	MBL fold metallo-hydrolase	WP_012421050.1
AMUC_RS11410	-0.137	0.047028	gene2276	Histidinol-phosphate aminotransferase	WP_012421161.1
AMUC_RS00890	-0.138	0.010129	gene176	Cell division protein FtsA	WP_051729163.1
AMUC_RS03205	-0.138	0.006942	gene639	Pyridoxal-5'-phosphate-dependent protein subunit beta	WP_012419637.1
AMUC_RS01830	-0.139	0.008499	gene363	DNA modification methylase	WP_012419380.1
AMUC_RS09470	-0.140	0.036634	gene1886	Hypothetical protein	WP_012420807.1
AMUC_RS07555	-0.142	0.001607	gene1501	Capsular polysaccharide biosynthesis protein	WP_012420451.1
AMUC_RS03750	-0.142	0.006901	gene747	Hypothetical protein	WP_012419742.1
AMUC_RS11035	-0.144	0.003484	gene2200	Aldehyde dehydrogenase	WP_012421090.1
AMUC_RS09075	-0.145	0.015304	gene1807	Tryptophan synthase subunit beta	WP_012420733.1
AMUC_RS08025	-0.145	0.01978	gene1596	Hypothetical protein	WP_052294469.1
AMUC_RS05495	-0.145	0.045917	gene1095	Disulfide bond chaperone	WP_012420066.1
AMUC_RS08830	-0.145	0.026312	gene1758	Leucine--tRNA ligase	WP_012420688.1
AMUC_RS06785	-0.146	0.007313	gene1349	PEP-CTERM domain protein	WP_031930890.1
AMUC_RS04020	-0.146	0.0024	gene801	Phospho-2-dehydro-3-deoxyheptonate aldolase	WP_012419795.1
AMUC_RS04260	-0.146	0.003271	gene850	O-acetylhomoserine aminocarboxypropyltransferase	WP_042448774.1
AMUC_RS04050	-0.146	0.025088	gene807	Hypothetical protein	WP_031930489.1
AMUC_RS04645	-0.147	0.030616	gene926	Cyclic nucleotide-binding protein	WP_012419906.1

AMUC_RS02605	-0.148	0.029285	gene519	Three-deoxy-D-manno-octulosonic-acid transferase	WP_012419527.1
AMUC_RS06805	-0.148	0.004018	gene1353	Thioredoxin-disulfide reductase	WP_012420309.1
AMUC_RS05560	-0.148	0.020619	gene1108	Transporter	WP_012420080.1
AMUC_RS07975	-0.150	0.048548	gene1586	Guanylate kinase	WP_012420526.1
AMUC_RS03835	-0.152	0.001088	gene764	Phosphoglycerate dehydrogenase	WP_012419757.1
AMUC_RS10580	-0.154	0.01885	gene2110	PEP-CTERM domain protein	WP_012421007.1
AMUC_RS09355	-0.155	0.049504	gene1863	Hypothetical protein	WP_042448279.1
AMUC_RS08490	-0.155	0.025098	gene1690	Hypothetical protein	WP_012420623.1
AMUC_RS02240	-0.155	0.000669	gene446	OmpH family outer membrane protein	WP_022198432.1
AMUC_RS07460	-0.155	0.009633	gene1483	Hydrogenase accessory protein HypB	WP_012420437.1
AMUC_RS09020	-0.155	0.000929	gene1796	Dihydrolipoyl dehydrogenase	WP_012420722.1
AMUC_RS04255	-0.155	0.018421	gene849	PEP-CTERM domain protein	WP_042447757.1
AMUC_RS05500	-0.156	0.017734	gene1096	Peptidyl-prolyl cis-trans isomerase	WP_012420067.1
AMUC_RS05295	-0.157	0.029956	gene1057	Succinate dehydrogenase (or fumarate reductase) cytochrome b subunit%2C b558 family	WP_012420030.1
AMUC_RS11425	-0.158	0.008499	gene2279	Transcriptional regulator	WP_012421163.1
AMUC_RS02990	-0.158	0.008954	gene596	Hypothetical protein	WP_012419600.1
AMUC_RS10080	-0.158	0.000918	gene2010	Glutamyl-Q tRNA(Asp) synthetase	WP_012420916.1
AMUC_RS05630	-0.159	0.014474	gene1122	ATP-dependent Clp protease proteolytic subunit	WP_012420092.1
AMUC_RS01440	-0.159	0.004022	gene285	4-hydroxy-tetrahydrodipicolinate synthase	WP_012419316.1
AMUC_RS09260	-0.159	0.004598	gene1844	Threonine--tRNA ligase	WP_012420764.1
AMUC_RS03915	-0.160	0.027239	gene780	Class II fructose-bisphosphate aldolase	WP_012419773.1
AMUC_RS02590	-0.160	0.038699	gene516	Phosphopantothenoylcysteine decarboxylase	WP_012419524.1
AMUC_RS12020	-0.160	0.006873	gene1337	Inner membrane insertion protein	WP_012420294.1
AMUC_RS04355	-0.161	0.003957	gene868	ATP-dependent RNA helicase HrpA	WP_052294440.1
AMUC_RS02985	-0.161	0.010168	gene595	Biopolymer transporter ExbD/TolR	WP_012419599.1
AMUC_RS07680	-0.161	0.004095	gene1527	Hypothetical protein	WP_042448085.1
AMUC_RS09700	-0.162	0.003392	gene1932	PUA domain containing protein	WP_012420850.1
AMUC_RS09175	-0.163	0.029347	gene1827	Hypothetical protein	WP_012420750.1
AMUC_RS02890	-0.164	0.010448	gene576	Gamma-glutamyl-phosphate reductase	WP_012419581.1
AMUC_RS03320	-0.164	0.025952	gene662	Hypothetical protein	WP_012419661.1
AMUC_RS04305	-0.165	0.001471	gene858	Lysine--tRNA ligase	WP_012419847.1
AMUC_RS10840	-0.166	0.005856	gene2161	Thymidylate synthase	WP_012421056.1
AMUC_RS02530	-0.166	0.006868	gene504	RNA polymerase sigma factor RpoE	WP_012419512.1
AMUC_RS02610	-0.167	0.005353	gene520	Methyltransferase	WP_012419528.1
AMUC_RS09975	-0.169	0.008469	gene1989	Peptide deformylase	WP_012420895.1
AMUC_RS08415	-0.170	0.001298	gene1675	Citrate synthase	WP_012420608.1
AMUC_RS01420	-0.170	0.046152	gene281	Xylose isomerase	WP_012419312.1

AMUC_RS00150	-0.171	0.004311	gene29	ATPase AAA	WP_042448497.1
AMUC_RS07390	-0.174	0.011517	gene1469	4-hydroxy-3-methylbut-2-en-1-yl diphosphate synthase (flavodoxin)	WP_012420427.1
AMUC_RS02305	-0.174	0.00585	gene459	Glutamate-1-semialdehyde 2%2C1-aminomutase	WP_012419470.1
AMUC_RS09555	-0.177	0.00449	gene1903	Peptidase S1 and S6 chymotrypsin/Hap	WP_012420823.1
AMUC_RS02795	-0.179	0.008417	gene557	ATP synthase subunit B	WP_012419563.1
AMUC_RS06110	-0.180	0.000332	gene1216	Hypothetical protein	WP_012420184.1
AMUC_RS06390	-0.180	0.04025	gene1272	Helicase c2	WP_012420237.1
AMUC_RS05640	-0.181	0.000128	gene1124	Outer membrane protein assembly factor BamA	WP_012420094.1
AMUC_RS00160	-0.181	0.027001	gene31	NAD-dependent epimerase/dehydratase family protein	WP_012419091.1
AMUC_RS03855	-0.181	0.001581	gene768	Transcriptional regulator	WP_012419761.1
AMUC_RS02910	-0.181	0.000256	gene580	GreA/GreB family elongation factor	WP_012419584.1
AMUC_RS07160	-0.182	0.00341	gene1424	Hypothetical protein	WP_042448019.1
AMUC_RS07625	-0.183	0.004546	gene1516	Hypothetical protein	WP_042448082.1
AMUC_RS04605	-0.183	0.004295	gene918	SAM-dependent methyltransferase	WP_012419898.1
AMUC_RS10860	-0.184	0.020945	gene2165	MATE efflux family protein	WP_012421060.1
AMUC_RS12200	-0.184	0.002034	gene2144	Phage-associated protein-like protein	WP_012421041.1
AMUC_RS10615	-0.184	0.00468	gene2117	Hypothetical protein	WP_012421014.1
AMUC_RS02740	-0.185	0.023704	gene546	Glycosyl transferase	WP_012419552.1
AMUC_RS09940	-0.185	0.025632	gene1982	Formate--tetrahydrofolate ligase	WP_042449223.1
AMUC_RS00800	-0.186	0.003192	gene159	Hypothetical protein	WP_042447473.1
AMUC_RS10795	-0.187	0.000206	gene2153	RNA helicase	WP_012421049.1
AMUC_RS00380	-0.187	0.002347	gene76	5-oxopent-3-ene-1%2C2%2C5-tricarboxylate decarboxylase	WP_012419124.1
AMUC_RS06020	-0.187	8.86E-05	gene1198	UDP-glucose 4-epimerase gale	WP_012420166.1
AMUC_RS00390	-0.187	0.000453	gene78	ATP/GTP-binding protein	WP_012419126.1
AMUC_RS04335	-0.187	0.008223	gene864	Imidazole glycerol phosphate synthase cyclase subunit	WP_012419852.1
AMUC_RS00900	-0.188	0.005969	gene178	Phosphomannomutase	WP_042448559.1
AMUC_RS04270	-0.189	0.003808	gene852	Radical SAM protein	WP_012419841.1
AMUC_RS08725	-0.189	0.006718	gene1737	ATP-dependent helicase HrpB	WP_012420668.1
AMUC_RS11565	-0.190	0.000736	gene2307	Arginine--tRNA ligase	WP_012421190.1
AMUC_RS01835	-0.191	0.001122	gene364	Hypothetical protein	WP_012419381.1
AMUC_RS04105	-0.192	0.037676	gene818	Acyltransferase	WP_012419812.1
AMUC_RS08975	-0.192	0.009988	gene1787	Nicotinate-nucleotide--dimethylbenzimidazole phosphoribosyltransferase	WP_012420714.1
AMUC_RS04110	-0.193	0.002686	gene819	Hypothetical protein	WP_012419813.1
AMUC_RS11300	-0.194	0.039026	gene2254	Hypothetical protein	WP_042448455.1
AMUC_RS11290	-0.194	8.17E-05	gene2252	Serine--tRNA ligase	WP_012421138.1

AMUC_RS02340	-0.195	0.000604	gene466	Bifunctional 3%2C4-dihydroxy-2-butanone 4-phosphate synthase/GTP cyclohydrolase II	WP_012419475.1
AMUC_RS10960	-0.195	0.007966	gene2185	Hypothetical protein	WP_012421077.1
AMUC_RS11045	-0.195	0.004744	gene2202	Hypothetical protein	WP_031931498.1
AMUC_RS01565	-0.196	0.006846	gene310	Signal transduction protein with efhand domain	WP_012419331.1
AMUC_RS06695	-0.196	0.000648	gene1331	Carbamoyl-phosphate synthase small chain	WP_012420290.1
AMUC_RS05130	-0.197	1.21E-05	gene1024	PEP-CTERM domain protein	WP_012419999.1
AMUC_RS08040	-0.197	7.27E-05	gene1599	Preprotein translocase subunit SecD	WP_012420538.1
AMUC_RS02235	-0.198	0.004868	gene445	Pantoate--beta-alanine ligase	WP_012419457.1
AMUC_RS05290	-0.198	0.027001	gene1056	Succinate dehydrogenase	WP_042448869.1
AMUC_RS04295	-0.201	0.001609	gene857	Protein-serine/threonine phosphatase	WP_012419846.1
AMUC_RS01200	-0.202	0.014678	gene237	PEP-CTERM domain protein	WP_012419273.1
AMUC_RS03930	-0.202	0.001555	gene783	Glucan endo-1%2C3-beta-D-glucosidase	WP_012419776.1
AMUC_RS00395	-0.203	0.001137	gene79	Aspartyl-tRNA amidotransferase subunit B	WP_012419127.1
AMUC_RS01540	-0.203	0.012254	gene305	Hypothetical protein	WP_052294415.1
AMUC_RS06375	-0.205	0.000526	gene1269	DNA topoisomerase IV subunit B	WP_012420234.1
AMUC_RS08045	-0.205	0.018699	gene1600	Signal transduction protein with efhand domain	WP_022197508.1
AMUC_RS08255	-0.207	0.000172	gene1643	Formate acetyltransferase	WP_012420579.1
AMUC_RS00110	-0.207	0.001577	gene21	MFS transporter	WP_012419082.1
AMUC_RS05670	-0.207	0.011054	gene1130	Acriflavin resistance protein	WP_012420100.1
AMUC_RS00210	-0.208	0.031397	gene41	Amino acid transporter	WP_042447403.1
AMUC_RS03765	-0.208	0.02625	gene750	Hypothetical protein	WP_012419745.1
AMUC_RS01980	-0.208	0.00538	gene394	Hypothetical protein	WP_012419408.1
AMUC_RS07905	-0.210	0.000526	gene1572	Imidazole glycerol phosphate synthase%2C glutamine amidotransferase subunit	WP_012420512.1
AMUC_RS08635	-0.211	0.029927	gene1719	Riboflavin synthase subunit alpha	WP_012420653.1
AMUC_RS06700	-0.212	0.000765	gene1332	Carbamoyl phosphate synthase large subunit	WP_012420291.1
AMUC_RS08590	-0.214	0.010737	gene1710	NADH dehydrogenase	WP_012420644.1
AMUC_RS11220	-0.215	0.00196	gene2238	Methionine--tRNA ligase	WP_012421126.1
AMUC_RS07950	-0.215	0.000695	gene1581	Hypothetical protein	WP_012420521.1
AMUC_RS07765	-0.216	0.001244	gene1544	Bifunctional ornithine acetyltransferase/N-acetylglutamate synthase	WP_012420485.1
AMUC_RS08245	-0.216	0.000376	gene1641	Hypothetical protein	WP_042448154.1
AMUC_RS11095	-0.217	0.000109	gene2213	Capsular polysaccharide biosynthesis protein	WP_012421101.1
AMUC_RS04740	-0.217	0.01108	gene945	Heavy metal translocating P-type ATPase	WP_012419924.1
AMUC_RS01600	-0.218	0.00198	gene317	Integration host factor subunit beta	WP_012419338.1
AMUC_RS00980	-0.218	0.000683	gene194	CTP synthetase	WP_012419229.1
AMUC_RS04730	-0.219	0.000242	gene943	Elongation factor P	WP_012419922.1

AMUC_RS01175	-0.219	0.000101	gene232	dTDP-glucose 4,6-dehydratase	WP_012419268.1
AMUC_RS02465	-0.219	0.008914	gene491	Glycine cleavage system protein H	WP_012419500.1
AMUC_RS05570	-0.219	0.007966	gene1110	DNA-directed RNA polymerase subunit beta	
AMUC_RS04040	-0.222	0.008179	gene805	Bifunctional riboflavin kinase/FMN adenylyltransferase	WP_012419799.1
AMUC_RS09615	-0.222	0.044937	gene1915	Aldo/keto reductase	WP_012420834.1
AMUC_RS02905	-0.223	8.6E-06	gene579	Transketolase	WP_042447629.1
AMUC_RS00170	-0.223	0.020695	gene33	Nucleotidyltransferase	WP_042448500.1
AMUC_RS03195	-0.224	0.000103	gene637	Hypothetical protein	WP_042447661.1
AMUC_RS01515	-0.226	0.000158	gene300	Phosphate acyltransferase	WP_012419323.1
AMUC_RS02570	-0.226	0.000895	gene512	Polyphosphate kinase	WP_031930275.1
AMUC_RS04080	-0.227	0.003139	gene813	ADP-L-glycero-D-mannoheptose-6-epimerase	WP_012419807.1
AMUC_RS04795	-0.227	0.002599	gene956	Pyruvate dehydrogenase	WP_012419935.1
AMUC_RS04010	-0.227	0.01069	gene799	Hypothetical protein	WP_012419793.1
AMUC_RS08825	-0.228	0.004432	gene1757	Ribulose-phosphate 3-epimerase	WP_012420687.1
AMUC_RS04160	-0.229	0.000109	gene829	Hypothetical protein	WP_052294436.1
AMUC_RS05985	-0.230	0.000274	gene1191	Sulfatase	WP_012420159.1
AMUC_RS05725	-0.231	0.005988	gene1141	Adenosylhomocysteinase	WP_012420110.1
AMUC_RS01270	-0.232	0.01356	gene251	Hypothetical protein	WP_012419286.1
AMUC_RS05585	-0.232	0.046984	gene1113	50S ribosomal protein L1	WP_012420085.1
AMUC_RS01905	-0.232	0.009064	gene379	Hypothetical protein	WP_042447558.1
AMUC_RS01660	-0.233	7.66E-05	gene329	Valine--tRNA ligase	WP_012419348.1
AMUC_RS05625	-0.233	1.27E-05	gene1121	ATP-dependent Clp protease ATP-binding subunit ClpX	WP_012420091.1
AMUC_RS09310	-0.233	3.12E-05	gene1854	Hypothetical protein	WP_012420775.1
AMUC_RS05270	-0.233	0.036895	gene1052	Hypothetical protein	WP_042447850.1
AMUC_RS01860	-0.234	0.003637	gene370	Hypothetical protein	WP_012419386.1
AMUC_RS07825	-0.234	8.15E-06	gene1556	Molecular chaperone DnaJ	WP_012420497.1
AMUC_RS11980	-0.235	0.00196	gene1178	Prolipoprotein diacylglyceryl transferase	WP_012420145.1
AMUC_RS05565	-0.235	0.01013	gene1109	DNA-directed RNA polymerase subunit beta'	WP_012420081.1
AMUC_RS08685	-0.236	3.73E-08	gene1729	RNA polymerase sigma factor RpoD	WP_012420661.1
AMUC_RS04360	-0.237	6.99E-05	gene869	Acetolactate synthase small subunit	WP_012419857.1
AMUC_RS01040	-0.237	0.004327	gene206	Hypothetical protein	WP_042447502.1
AMUC_RS01710	-0.239	0.00315	gene339	30S ribosomal protein S19	WP_012419357.1
AMUC_RS11105	-0.239	0.000868	gene2215	Hypothetical protein	
AMUC_RS06735	-0.241	0.018998	gene1339	Ribonuclease P protein component	WP_051729509.1
AMUC_RS00860	-0.242	1.24E-06	gene171	Saccharopine dehydrogenase	WP_012419207.1
AMUC_RS00820	-0.243	0.009361	gene163	Hypothetical protein	WP_042447481.1
AMUC_RS00545	-0.243	0.001277	gene109	Hydroxymethylbilane synthase	WP_012419150.1
AMUC_RS00435	-0.244	0.008453	gene87	Hypothetical protein	WP_012419134.1
AMUC_RS08315	-0.245	0.013804	gene1655	Hypothetical protein	WP_012420590.1

AMUC_RS01580	-0.245	0.01087	gene313	tRNA (guanine-N(7)-)-methyltransferase	WP_012419334.1
AMUC_RS00115	-0.245	0.001518	gene22	RNA polymerase sigma-54 factor	WP_042447387.1
AMUC_RS11775	-0.245	0.012299	gene427	Hypothetical protein	WP_052294423.1
AMUC_RS02680	-0.247	0.000199	gene534	Fe-S cluster assembly protein SufB	WP_012419541.1
AMUC_RS08070	-0.248	6.28E-06	gene1605	Malonyl coa-acyl carrier protein transacylase	WP_012420543.1
AMUC_RS07930	-0.248	9.14E-06	gene1577	Hypothetical protein	WP_042448123.1
AMUC_RS04565	-0.249	0.010076	gene910	Zinc finger SWIM domain-containing protein	WP_012419890.1
AMUC_RS10540	-0.249	0.00342	gene2102	Leucyl/phenylalanyl-tRNA--protein transferase	WP_012421000.1
AMUC_RS03820	-0.250	0.000829	gene761	Aminotransferase	WP_012419755.1
AMUC_RS05550	-0.250	1.05E-06	gene1106	Elongation factor 4	WP_012420077.1
AMUC_RS06885	-0.250	2.56E-06	gene1369	DNA gyrase subunit B	WP_012420325.1
AMUC_RS06620	-0.250	0.000107	gene1316	30S ribosomal protein S9	WP_012420275.1
AMUC_RS09675	-0.250	0.038533	gene1927	MATE family efflux transporter	WP_012420845.1
AMUC_RS06610	-0.252	3.09E-05	gene1314	Translation initiation factor IF-1	WP_042448973.1
AMUC_RS07630	-0.252	0.016904	gene1517	Hypothetical protein	WP_012420463.1
AMUC_RS07520	-0.253	0.02283	gene1494	Molecular chaperone GroEL	WP_042448069.1
AMUC_RS07075	-0.253	0.000126	gene1407	Hypothetical protein	WP_012420362.1
AMUC_RS07560	-0.254	3.08E-06	gene1502	Polysaccharide export protein	WP_012420452.1
AMUC_RS09160	-0.255	1.43E-06	gene1824	Histidinol dehydrogenase	WP_012420747.1
AMUC_RS03625	-0.256	0.010527	gene722	Isopropylmalate isomerase	WP_012419719.1
AMUC_RS10555	-0.258	2.17E-07	gene2105	Glucose-6-phosphate isomerase	WP_012421002.1
AMUC_RS03670	-0.258	0.023627	gene731	30S ribosomal protein S2	WP_012419726.1
AMUC_RS00930	-0.259	0.004023	gene184	Mammalian cell entry protein	WP_012419220.1
AMUC_RS05410	-0.259	3.01E-08	gene1078	Alpha-xylosidase	WP_012420049.1
AMUC_RS10000	-0.260	0.011915	gene1994	Hypothetical protein	WP_052294487.1
AMUC_RS09365	-0.261	9.44E-07	gene1865	Ornithine carbamoyltransferase	WP_012420786.1
AMUC_RS00560	-0.262	5.67E-05	gene112	GTP cyclohydrolase fole2	WP_012419153.1
AMUC_RS02655	-0.262	0.027309	gene529	tRNA threonylcarbamoyladenosine biosynthesis protein tsae	WP_012419536.1
AMUC_RS07115	-0.262	3.46E-07	gene1415	PEP-CTERM domain protein	WP_012420372.1
AMUC_RS01630	-0.263	0.012996	gene323	Hypothetical protein	WP_012419343.1
AMUC_RS00925	-0.263	0.004546	gene183	Organic solvent ABC transporter ATP-binding protein	WP_012419219.1
AMUC_RS04045	-0.263	6.99E-05	gene806	tRNA pseudouridine(55) synthase	WP_012419800.1
AMUC_RS01970	-0.263	0.008758	gene392	Hypothetical protein	WP_012419406.1
AMUC_RS07590	-0.264	0.00974	gene1508	Hypothetical protein	WP_042448074.1
AMUC_RS07580	-0.267	0.000338	gene1506	Phosphoglycerate kinase	WP_012420456.1
AMUC_RS02455	-0.267	3.68E-05	gene489	GTP-binding protein YchF	WP_012419498.1
AMUC_RS05790	-0.267	7.72E-07	gene1152	L-aspartate oxidase	WP_012420120.1
AMUC_RS02210	-0.268	0.000112	gene440	ATP-dependent dethiobiotin synthetase BioD	WP_012419452.1
AMUC_RS04350	-0.268	0.001299	gene867	Trka-N domain-containing protein	WP_012419855.1

AMUC_RS08260	-0.269	0.003445	gene1644	Hypothetical protein	WP_052294470.1
AMUC_RS06465	-0.269	4.41E-06	gene1287	ATP-dependent DNA helicase RecG	WP_042447962.1
AMUC_RS08710	-0.269	0.000145	gene1734	Tail-specific protease	WP_012420666.1
AMUC_RS11735	-0.269	0.0004	gene349	Hypothetical protein	WP_052294418.1
AMUC_RS02430	-0.269	0.002779	gene484	Epoxyqueuosine reductase	WP_012419493.1
AMUC_RS08845	-0.270	4.38E-06	gene1761	Carboxynorspermidine decarboxylase	WP_012420691.1
AMUC_RS04850	-0.270	1.16E-06	gene967	Uroporphyrin-III C-methyltransferase	WP_012419944.1
AMUC_RS01530	-0.275	0.00882	gene303	Histidine triad nucleotide-binding protein	WP_012419326.1
AMUC_RS02115	-0.275	0.000177	gene421	Hypothetical protein	WP_012419434.1
AMUC_RS04860	-0.276	2.65E-07	gene969	DNA topoisomerase III	WP_012419946.1
AMUC_RS07985	-0.279	0.001496	gene1588	tRNA glutamyl-Q synthetase	WP_012420528.1
AMUC_RS04015	-0.281	0.007048	gene800	Hypothetical protein	WP_042447740.1
AMUC_RS04640	-0.281	3.42E-05	gene925	Hypothetical protein	WP_012419905.1
AMUC_RS01680	-0.281	0.00585	gene333	30S ribosomal protein S17	WP_012419351.1
AMUC_RS08555	-0.282	0.000502	gene1703	Hypothetical protein	WP_012420636.1
AMUC_RS09325	-0.282	3.17E-06	gene1857	Glycerol-3-phosphate cytidiltransferase	WP_012420779.1
AMUC_RS11950	-0.283	0.000315	gene1038	RNP-1 like RNA-binding protein	WP_012420014.1
AMUC_RS00665	-0.283	0.017601	gene133	Hypothetical protein	WP_042447452.1
AMUC_RS09460	-0.283	0.001115	gene1884	Hypothetical protein	WP_012420805.1
AMUC_RS04125	-0.283	0.000176	gene822	SLC13 family permease	WP_012419816.1
AMUC_RS00815	-0.285	3.92E-08	gene162	Tyrosine--tRNA ligase	WP_012419199.1
AMUC_RS02545	-0.285	0.002175	gene507	Hypothetical protein	WP_012419515.1
AMUC_RS01715	-0.285	0.021003	gene340	50S ribosomal protein L2	WP_012419358.1
AMUC_RS06655	-0.286	9.37E-06	gene1323	Inositol monophosphatase	WP_012420282.1
AMUC_RS02300	-0.287	0.000427	gene458	30S ribosomal protein S21	WP_012419469.1
AMUC_RS01525	-0.288	0.001012	gene302	Hypothetical protein	WP_042447540.1
AMUC_RS10360	-0.289	1.74E-09	gene2067	Adenylosuccinate lyase	WP_012420968.1
AMUC_RS03740	-0.289	4.15E-05	gene745	PEP-CTERM domain protein	WP_042447709.1
AMUC_RS11270	-0.289	4.53E-05	gene2248	Hypothetical protein	WP_052294494.1
AMUC_RS09725	-0.291	1.45E-09	gene1937	Glucosamine-6-phosphate deaminase	WP_012420854.1
AMUC_RS08100	-0.291	0.004374	gene1611	Hypothetical protein	WP_042448135.1
AMUC_RS00660	-0.291	0.000951	gene132	Phosphoribosylaminoimidazole carboxylase	WP_012419174.1
AMUC_RS03745	-0.291	6.85E-06	gene746	Asparagine--tRNA ligase	WP_042448742.1
AMUC_RS03065	-0.292	1.11E-08	gene611	Argininosuccinate synthase	WP_012419612.1
AMUC_RS05575	-0.293	4.77E-06	gene1111	50S ribosomal protein L7/L12	WP_012420083.1
AMUC_RS01840	-0.294	6.99E-05	gene365	Hypothetical protein	WP_012419382.1
AMUC_RS07880	-0.296	1.04E-08	gene1567	Ribose-phosphate pyrophosphokinase	WP_012420508.1
AMUC_RS00420	-0.297	1.98E-05	gene84	Metallophosphoesterase	WP_042448517.1
AMUC_RS06860	-0.297	0.000802	gene1364	Aminopeptidase	WP_012420320.1
AMUC_RS07885	-0.298	8.14E-09	gene1568	50S ribosomal protein L25	WP_012420509.1

AMUC_RS04555	-0.298	4.63E-06	gene908	Sodium-independent anion transporter	WP_012419888.1
AMUC_RS01370	-0.299	8.15E-06	gene271	Marr family transcriptional regulator	WP_012419304.1
AMUC_RS02195	-0.299	1.58E-06	gene437	Ribonuclease PH	WP_042448654.1
AMUC_RS06775	-0.299	7.27E-05	gene1347	Farnesyl-diphosphate synthase	WP_012420304.1
AMUC_RS00995	-0.300	2.72E-05	gene197	Hypothetical protein	WP_012419232.1
AMUC_RS10980	-0.300	2.18E-06	gene2189	Hypothetical protein	WP_012421081.1
AMUC_RS09170	-0.301	1.98E-07	gene1826	Hypothetical protein	WP_012420749.1
AMUC_RS09390	-0.302	1.09E-11	gene1870	Peptidase M20	WP_042449168.1
AMUC_RS01755	-0.302	4.29E-08	gene348	2,3-bisphosphoglycerate-independent phosphoglycerate mutase	WP_012419366.1
AMUC_RS11180	-0.302	0.000894	gene2230	Hypothetical protein	WP_042448440.1
AMUC_RS08300	-0.303	0.000263	gene1652	MFS transporter	WP_012420588.1
AMUC_RS10205	-0.304	3.5E-08	gene2036	DEAD/DEAH box helicase	WP_012420940.1
AMUC_RS09830	-0.305	5.52E-05	gene1959	Phosphomethylpyrimidine synthase ThiC	WP_012420871.1
AMUC_RS02715	-0.307	0.026049	gene541	Hypothetical protein	WP_012419547.1
AMUC_RS11200	-0.307	3.06E-06	gene2234	Hypothetical protein	WP_042448443.1
AMUC_RS04475	-0.308	0.000107	gene892	Potassium transporter TrkH	WP_042448790.1
AMUC_RS07020	-0.308	0.013931	gene1396	Phosphohydrolase	WP_042448008.1
AMUC_RS05260	-0.310	0.002141	gene1050	Hypothetical protein	WP_042447845.1
AMUC_RS01785	-0.312	1.08E-05	gene354	1-deoxy-D-xylulose-5-phosphate synthase	WP_012419372.1
AMUC_RS06075	-0.313	0.033553	gene1209	Hypothetical protein	WP_042447926.1
AMUC_RS06685	-0.313	5.87E-06	gene1329	GDP-fucose synthetase	WP_012420288.1
AMUC_RS11550	-0.314	2.85E-09	gene2304	Oxidoreductase	WP_012421187.1
AMUC_RS10490	-0.314	4.19E-07	gene2092	Homoserine dehydrogenase	WP_012420991.1
AMUC_RS09640	-0.315	0.002303	gene1920	Aldo/keto reductase	WP_012420839.1
AMUC_RS02415	-0.315	1.66E-08	gene481	30S ribosomal protein S4	WP_012419490.1
AMUC_RS01480	-0.317	3.15E-07	gene293	Hypothetical protein	WP_042447535.1
AMUC_RS07820	-0.317	1.05E-06	gene1555	Nucleotide exchange factor GrpE	WP_012420496.1
AMUC_RS03720	-0.318	0.046164	gene741	HxlR family transcriptional regulator	WP_012419736.1
AMUC_RS06380	-0.319	1.9E-05	gene1270	4-(cytidine 5'-diphospho)-2-C-methyl-D-erythritol kinase	WP_012420235.1
AMUC_RS07780	-0.322	4.85E-05	gene1547	PEP-CTERM domain protein	WP_012420488.1
AMUC_RS00685	-0.323	7.16E-07	gene137	Hypothetical protein	WP_012419178.1
AMUC_RS10060	-0.324	0.000137	gene2006	Thymidylate kinase	WP_012420912.1
AMUC_RS02045	-0.325	7.76E-08	gene407	Sigma-54-dependent Fis family transcriptional regulator	WP_012419420.1
AMUC_RS01035	-0.325	5.52E-05	gene205	RDD domain-containing protein	WP_012419240.1
AMUC_RS05635	-0.326	7.6E-07	gene1123	Trigger factor	WP_042447874.1
AMUC_RS10495	-0.329	3.1E-07	gene2093	Aspartate kinase	WP_012420992.1
AMUC_RS03770	-0.329	0.00011	gene751	Hypothetical protein	WP_042447712.1
AMUC_RS02735	-0.330	0.015013	gene545	Hypothetical protein	WP_012419551.1
AMUC_RS08335	-0.331	1.64E-06	gene1659	RIP metalloprotease RseP	WP_012420594.1

AMUC_RS01180	-0.331	4.37E-16	gene233	6-phosphofructokinase	WP_012419269.1
AMUC_RS02085	-0.332	1.79E-14	gene415	DNA polymerase III subunit alpha	WP_012419428.1
AMUC_RS07910	-0.332	1.14E-06	gene1573	30S ribosomal protein S6	WP_012420513.1
AMUC_RS02140	-0.333	0.000703	gene426	Maltose acetyltransferase	
AMUC_RS01330	-0.333	0.010103	gene263	Arsc family transcriptional regulator	WP_012419295.1
AMUC_RS08225	-0.334	0.0001	gene1637	Hypothetical protein	WP_042448151.1
AMUC_RS09665	-0.335	0.001412	gene1925	Alpha-amylase	WP_012420844.1
AMUC_RS10235	-0.336	6.21E-12	gene2042	UDP-3-O-[3-hydroxymyristoyl] N-acetylglucosamine deacetylase	WP_012420945.1
AMUC_RS09025	-0.336	0.000869	gene1797	Nucleotidyltransferase	WP_012420723.1
AMUC_RS03985	-0.337	4.98E-05	gene794	Phosphotyrosine protein phosphatase	WP_012419787.1
AMUC_RS05580	-0.337	0.000521	gene1112	50S ribosomal protein L10	WP_012420084.1
AMUC_RS02230	-0.338	5.84E-06	gene444	ABC transporter ATP-binding protein	WP_012419456.1
AMUC_RS00680	-0.339	9.99E-08	gene136	Agmatine deiminase	WP_012419177.1
AMUC_RS06335	-0.340	0.007135	gene1261	Hypothetical protein	WP_052294459.1
AMUC_RS07380	-0.340	0.003057	gene1467	Dihydropteroate synthase	WP_012420425.1
AMUC_RS06435	-0.340	1.96E-08	gene1281	Bifunctional phosphoribosylaminoimidazolecarboxamide formyltransferase/inosine monophosphate cyclohydrolase	WP_012420246.1
AMUC_RS08445	-0.341	8.02E-05	gene1681	Hypothetical protein	WP_042448186.1
AMUC_RS06570	-0.341	0.000166	gene1307	Nucleoid-associated protein	WP_012420267.1
AMUC_RS02410	-0.344	1.19E-05	gene480	Haloacid dehalogenase	WP_012419489.1
AMUC_RS10325	-0.346	0.003208	gene2060	Acyltransferase	WP_012420962.1
AMUC_RS08800	-0.346	0.003338	gene1752	30S ribosomal protein S20	WP_012420682.1
AMUC_RS07980	-0.347	5.27E-08	gene1587	TrkA-C domain-containing protein	WP_012420527.1
AMUC_RS03080	-0.348	2.37E-14	gene615	Triose-phosphate isomerase	WP_012419614.1
AMUC_RS08790	-0.349	1.33E-10	gene1750	4-hydroxy-3-methylbut-2-enyl diphosphate reductase	WP_012420681.1
AMUC_RS11760	-0.351	6.93E-06	gene393	Hypothetical protein	WP_012419407.1
AMUC_RS00550	-0.351	2.08E-06	gene110	Glutamyl-tRNA reductase	WP_012419151.1
AMUC_RS08390	-0.351	1.77E-05	gene1670	Methylated-DNA--protein-cysteine methyltransferase	WP_012420603.1
AMUC_RS08705	-0.352	3E-06	gene1733	Serine hydroxymethyltransferase	WP_042449109.1
AMUC_RS01030	-0.353	7.68E-11	gene204	Acyl-[acyl-carrier-protein]--UDP-N-acetylglucosamine O-acyltransferase	WP_012419239.1
AMUC_RS08650	-0.353	0.005988	gene1722	Outer membrane autotransporter barrel domain-containing protein	WP_012420655.1
AMUC_RS04470	-0.354	0.00161	gene891	Potassium-transporting ATPase subunit A	WP_012419878.1
AMUC_RS10160	-0.355	4.56E-05	gene2027	Hypothetical protein	WP_012420931.1
AMUC_RS10090	-0.355	1.41E-08	gene2012	UDP-diphosphatase	WP_012420918.1

AMUC_RS02290	-0.355	7.48E-10	gene456	Bifunctional heptose 7-phosphate kinase/heptose 1-phosphate adenyltransferase	WP_012419468.1
AMUC_RS04455	-0.356	3.66E-09	gene888	Two-component sensor histidine kinase	WP_012419875.1
AMUC_RS05875	-0.357	4.44E-07	gene1169	Heavy metal translocating P-type ATPase	WP_012420136.1
AMUC_RS01535	-0.359	1.41E-08	gene304	Translation factor Sua5	WP_012419327.1
AMUC_RS08190	-0.359	4.57E-06	gene1630	Anthranilate synthase	WP_042448145.1
AMUC_RS11110	-0.361	1.74E-10	gene2216	Glycosyl transferase	WP_012421104.1
AMUC_RS02640	-0.361	2.13E-10	gene526	Alpha-1%2C3-galactosidase	WP_012419533.1
AMUC_RS06080	-0.362	0.009792	gene1210	Hypothetical protein	WP_012420178.1
AMUC_RS10465	-0.363	1.31E-09	gene2088	Methionine synthase	WP_012420988.1
AMUC_RS11265	-0.364	9.98E-09	gene2247	Orotate phosphoribosyltransferase	WP_012421133.1
AMUC_RS07280	-0.366	5.77E-05	gene1447	Hypothetical protein	WP_042448029.1
AMUC_RS08285	-0.367	0.000176	gene1649	Hypothetical protein	
AMUC_RS06560	-0.367	1.81E-11	gene1305	Hypothetical protein	WP_052294500.1
AMUC_RS05750	-0.367	9.08E-05	gene1146	Hypothetical protein	WP_042447893.1
AMUC_RS00780	-0.368	4.65E-06	gene155	Chromosome partitioning protein ParB	WP_012419192.1
AMUC_RS04000	-0.369	7.15E-05	gene797	Maltose acetyltransferase	WP_031930480.1
AMUC_RS10215	-0.369	7.39E-12	gene2038	Type I restriction-modification protein subunit M	WP_012420942.1
AMUC_RS02395	-0.370	1.23E-05	gene477	Hypothetical protein	WP_012419486.1
AMUC_RS02095	-0.370	0.001581	gene417	Thiamine phosphate synthase	WP_052294422.1
AMUC_RS12180	-0.371	2.03E-06	gene2037	Restriction modification system DNA specificity domain	WP_012420941.1
AMUC_RS06550	-0.372	1.44E-12	gene1303	Orotidine 5'-phosphate decarboxylase	WP_012420263.1
AMUC_RS07405	-0.376	0.000813	gene1472	Hypothetical protein	WP_012420429.1
AMUC_RS03950	-0.376	9.3E-12	gene787	GTPase ObgE	WP_022197812.1
AMUC_RS07575	-0.379	2.15E-06	gene1505	Type I glyceraldehyde-3-phosphate dehydrogenase	WP_012420455.1
AMUC_RS10330	-0.380	1.42E-09	gene2061	Hypothetical protein	WP_031931384.1
AMUC_RS05960	-0.383	1.94E-07	gene1186	Dihydroxy-acid dehydratase	WP_012420152.1
AMUC_RS09605	-0.384	0.010281	gene1913	2%2C5-diketo-D-gluconic acid reductase	WP_012420832.1
AMUC_RS07570	-0.384	1.86E-09	gene1504	Holliday junction ATP-dependent DNA helicase RuvA	WP_042449043.1
AMUC_RS12150	-0.385	7.43E-10	gene1960	Thioredoxin domain	WP_012420872.1
AMUC_RS09920	-0.385	5.56E-10	gene1978	Metallophosphoesterase	WP_012420884.1
AMUC_RS04930	-0.386	2.58E-07	gene983	ATP--cobalamin adenosyltransferase	WP_012419960.1
AMUC_RS07760	-0.387	9.81E-06	gene1543	N-acetyl-gamma-glutamyl-phosphate reductase	WP_012420484.1
AMUC_RS07270	-0.389	3.46E-07	gene1445	Hypothetical protein	WP_012420404.1
AMUC_RS01635	-0.391	1.52E-06	gene324	MotA/TolQ/ExbB proton channel	WP_012419344.1
AMUC_RS06290	-0.391	1.97E-05	gene1252	Ketol-acid reductoisomerase	WP_012420218.1

AMUC_RS05350	-0.394	9.1E-14	gene1067	ABC transporter ATP-binding protein	WP_012420039.1
AMUC_RS10435	-0.395	0.015431	gene2082	Aminoacyl-tRNA deacylase	WP_012420983.1
AMUC_RS02005	-0.398	2.58E-10	gene399	Hypothetical protein	WP_012419413.1
AMUC_RS08850	-0.398	1.68E-14	gene1762	Arginine decarboxylase	WP_012420692.1
AMUC_RS07365	-0.398	1.3E-18	gene1464	Alanine--tRNA ligase	WP_012420422.1
AMUC_RS07455	-0.399	3.24E-12	gene1482	Histidine--tRNA ligase	WP_012420436.1
AMUC_RS02335	-0.401	4.53E-15	gene465	Glutamine--tRNA ligase	WP_012419474.1
AMUC_RS09610	-0.402	0.009555	gene1914	Alpha/beta hydrolase	WP_012420833.1
AMUC_RS10790	-0.404	0.001554	gene2152	Crossover junction endodeoxyribonuclease ruvc	WP_012421048.1
AMUC_RS04610	-0.404	6.09E-05	gene919	Hypothetical protein	WP_012419899.1
AMUC_RS10035	-0.405	1.11E-09	gene2001	Isocitrate dehydrogenase (NADP(+))	WP_012420908.1
AMUC_RS08410	-0.408	6.67E-07	gene1674	tRNA threonylcarbamoyladenosine biosynthesis protein tsab	WP_042448182.1
AMUC_RS05355	-0.410	3.66E-09	gene1068	Hypothetical protein	WP_012420040.1
AMUC_RS02930	-0.412	6.07E-14	gene584	Phosphoribosylformylglycinamidine synthase	WP_012419588.1
AMUC_RS08425	-0.413	4.46E-07	gene1677	Helicase	WP_012420610.1
AMUC_RS09530	-0.414	3.09E-11	gene1898	Hypothetical protein	WP_042448288.1
AMUC_RS07415	-0.414	7.33E-09	gene1474	Hypothetical protein	WP_042448050.1
AMUC_RS04100	-0.416	8.78E-05	gene817	Hypothetical protein	WP_012419811.1
AMUC_RS05020	-0.416	6.77E-07	gene1002	30S ribosomal protein S5	WP_031930630.1
AMUC_RS05610	-0.417	2.11E-07	gene1118	Elongation factor Tu	WP_012420089.1
AMUC_RS09595	-0.419	0.005923	gene1911	Cystathionine beta-lyase	WP_012420830.1
AMUC_RS02920	-0.421	7.57E-08	gene582	Hypothetical protein	WP_012419586.1
AMUC_RS06095	-0.422	1.21E-05	gene1213	Hypothetical protein	WP_012420181.1
AMUC_RS07745	-0.423	0.002495	gene1540	Peptide ABC transporter ATP-binding protein	WP_042449053.1
AMUC_RS11730	-0.425	0.010168	gene325	Hypothetical protein	WP_052294417.1
AMUC_RS02585	-0.428	1.77E-07	gene515	Hypothetical protein	WP_052294426.1
AMUC_RS09400	-0.429	1.07E-07	gene1872	5-formyltetrahydrofolate cyclo-ligase	WP_012420793.1
AMUC_RS07450	-0.431	6.23E-12	gene1481	Aspartate--tRNA ligase	WP_012420435.1
AMUC_RS08615	-0.432	1.85E-16	gene1715	NADH-quinone oxidoreductase subunit D	WP_012420649.1
AMUC_RS05375	-0.436	0.012617	gene1072	Short-chain dehydrogenase	WP_012420043.1
AMUC_RS11510	-0.439	8.36E-14	gene2296	NADH (or F420H2) dehydrogenase	WP_012421179.1
AMUC_RS04755	-0.439	2.27E-08	gene948	WecB/TagA/CpsF family glycosyl transferase	WP_012419927.1
AMUC_RS03875	-0.441	8.29E-07	gene772	Trna preq1(34) S-adenosylmethionine ribosyltransferase-isomerase QueA	WP_012419765.1
AMUC_RS08345	-0.441	1.23E-21	gene1661	PEP-CTERM domain protein	WP_042448170.1
AMUC_RS01365	-0.443	4.04E-09	gene270	Peptide chain release factor 2	WP_012419303.1
AMUC_RS06875	-0.445	9.74E-15	gene1367	Hypothetical protein	WP_042448001.1
AMUC_RS01725	-0.445	0.000722	gene342	50S ribosomal protein L4	WP_012419360.1
AMUC_RS06030	-0.445	1.46E-11	gene1200	Hypothetical protein	WP_012420168.1

AMUC_RS04485	-0.447	4.52E-20	gene894	Cupin	WP_042448792.1
AMUC_RS11140	-0.449	1.41E-07	gene2222	Hypothetical protein	WP_012421110.1
AMUC_RS06155	-0.453	0.000212	gene1225	Osmosensitive K channel His kinase sensor	WP_012420194.1
AMUC_RS01375	-0.454	5.88E-09	gene272	30S ribosomal protein S1	WP_012419305.1
AMUC_RS04650	-0.456	7.54E-12	gene927	Anion transporter	WP_012419907.1
AMUC_RS01120	-0.456	1.58E-09	gene221	50S ribosomal protein L19	WP_012419257.1
AMUC_RS10785	-0.456	1.55E-07	gene2151	Hypothetical protein	WP_012421047.1
AMUC_RS04490	-0.457	2.56E-25	gene895	Marr family transcriptional regulator	WP_012419882.1
AMUC_RS08320	-0.458	1.09E-09	gene1656	Excinuclease ABC subunit B	WP_012420591.1
AMUC_RS09105	-0.458	2.18E-06	gene1813	Hypothetical protein	WP_012420739.1
AMUC_RS00450	-0.459	2.18E-07	gene90	NUDIX hydrolase	WP_012419136.1
AMUC_RS08325	-0.461	2.32E-10	gene1657	50S ribosomal protein L35	WP_012420592.1
AMUC_RS09590	-0.462	0.000101	gene1910	Hypothetical protein	WP_012420829.1
AMUC_RS02080	-0.464	2.97E-06	gene414	GNAT family acetyltransferase	WP_012419427.1
AMUC_RS05025	-0.466	1.97E-09	gene1003	50S ribosomal protein L15	WP_012419978.1
AMUC_RS09210	-0.467	7.27E-12	gene1834	Hypothetical protein	WP_042448259.1
AMUC_RS11705	-0.472	1.84E-09	gene214	Peptidase A8 signal peptidase II	WP_012419249.1
AMUC_RS10865	-0.474	9.65E-18	gene2166	Hypothetical protein	WP_012421061.1
AMUC_RS05870	-0.474	7.54E-15	gene1168	Hypothetical protein	
AMUC_RS08875	-0.475	0.004022	gene1767	Hypothetical protein	WP_042448212.1
AMUC_RS01720	-0.476	0.000201	gene341	50S ribosomal protein L23	WP_012419359.1
AMUC_RS06135	-0.478	7.38E-13	gene1221	Hypothetical protein	WP_042447936.1
AMUC_RS06430	-0.478	9.12E-12	gene1280	50S ribosomal protein L9	WP_012420245.1
AMUC_RS02915	-0.479	2.53E-12	gene581	Phosphoribosylaminoimidazolesucci nocarboxamide synthase	WP_012419585.1
AMUC_RS06295	-0.482	7.23E-09	gene1253	Hypothetical protein	WP_052294499.1
AMUC_RS08065	-0.486	3.29E-08	gene1604	50S ribosomal protein L17	WP_012420542.1
AMUC_RS04235	-0.486	6.46E-12	gene845	3-isopropylmalate dehydrogenase	WP_012419834.1
AMUC_RS10335	-0.496	2.52E-05	gene2062	Phosphoribosyltransferase	WP_012420964.1
AMUC_RS00410	-0.497	8.07E-24	gene82	Tryptophan--tRNA ligase	WP_012419130.1
AMUC_RS02495	-0.498	1.02E-24	gene497	Hypothetical protein	WP_052294425.1
AMUC_RS06305	-0.502	0.000459	gene1255	Hypothetical protein	WP_042447951.1
AMUC_RS08105	-0.507	2.4E-15	gene1612	Hypothetical protein	WP_012420550.1
AMUC_RS10030	-0.508	0.002686	gene2000	Hypothetical protein	WP_012420907.1
AMUC_RS01340	-0.509	0.036096	gene265	Hypothetical protein	WP_042447522.1
AMUC_RS03865	-0.511	2.07E-07	gene770	Sodium/hydrogen exchanger	WP_012419763.1
AMUC_RS05255	-0.511	9.47E-05	gene1049	D-aminoacyl-tRNA deacylase	WP_012420025.1
AMUC_RS00370	-0.512	0.000357	gene74	Hypothetical protein	WP_052294407.1
AMUC_RS01910	-0.513	1.46E-12	gene380	OsmC family protein	WP_012419396.1
AMUC_RS08550	-0.514	3.6E-10	gene1702	Putative Fe-S cluster assembly protein SufT	WP_012420635.1
AMUC_RS07070	-0.515	2.62E-11	gene1406	Hypothetical protein	WP_052294464.1
AMUC_RS02870	-0.516	1.23E-20	gene572	Hypothetical protein	WP_042447621.1
AMUC_RS02745	-0.516	1.46E-08	gene547	N-acetyltransferase GCN5	WP_012419553.1

AMUC_RS06615	-0.517	6.69E-19	gene1315	50S ribosomal protein L13	WP_012420274.1
AMUC_RS01010	-0.517	5.83E-13	gene200	Hypothetical protein	WP_042447500.1
AMUC_RS02425	-0.517	1.18E-28	gene483	30S ribosomal protein S13	WP_012419492.1
AMUC_RS06280	-0.518	0.03975	gene1250	Lipocalin	WP_012420217.1
AMUC_RS02665	-0.528	1.28E-11	gene531	50S ribosomal protein L27	WP_012419538.1
AMUC_RS07750	-0.528	0.016048	gene1541	Amino acid ABC transporter permease	WP_042449055.1
AMUC_RS05590	-0.530	1.9E-11	gene1114	50S ribosomal protein L11	WP_012420086.1
AMUC_RS07480	-0.532	2.58E-11	gene1487	Hydrogenase expression/formation protein hype	WP_012420441.1
AMUC_RS03655	-0.533	1.41E-07	gene728	Peptide ABC transporter ATPase	WP_012419724.1
AMUC_RS07470	-0.535	0.009792	gene1485	Hydrogenase assembly protein HupF	WP_012420439.1
AMUC_RS01740	-0.536	2.56E-06	gene345	Elongation factor G	WP_012419363.1
AMUC_RS04035	-0.537	1.14E-09	gene804	Aspartate decarboxylase	WP_012419798.1
AMUC_RS05720	-0.538	3.78E-06	gene1140	S-adenosylmethionine synthase	WP_012420109.1
AMUC_RS04005	-0.541	2.3E-08	gene798	Phosphoglycolate phosphatase	WP_012419791.1
AMUC_RS08140	-0.545	1.46E-18	gene1619	DNA methylase	WP_012420557.1
AMUC_RS06195	-0.552	2.28E-05	gene1233	Hypothetical protein	WP_052294456.1
AMUC_RS02420	-0.553	7.37E-15	gene482	30S ribosomal protein S11	WP_012419491.1
AMUC_RS02285	-0.562	1.2E-12	gene455	Phosphoheptose isomerase	WP_012419467.1
AMUC_RS01310	-0.565	9.12E-37	gene259	L-threonine 3-dehydrogenase	WP_012419291.1
AMUC_RS03125	-0.565	1.31E-08	gene623	Hypothetical protein	WP_012419622.1
AMUC_RS01350	-0.568	2.17E-13	gene267	Hypothetical protein	WP_042447523.1
AMUC_RS10775	-0.573	0.000263	gene2149	NUDIX domain-containing protein	WP_052294492.1
AMUC_RS04200	-0.575	0.000106	gene838	Hypothetical protein	WP_052294437.1
AMUC_RS05245	-0.575	2.52E-16	gene1047	Hypothetical protein	WP_012420023.1
AMUC_RS07675	-0.577	0.006033	gene1526	Hypothetical protein	WP_012420470.1
AMUC_RS04265	-0.582	5.47E-18	gene851	Radical SAM protein	WP_012419840.1
AMUC_RS09890	-0.583	8.33E-27	gene1972	Alanine racemase	WP_042448328.1
AMUC_RS03260	-0.585	4.81E-10	gene650	Flavin reductase	WP_042448704.1
AMUC_RS08145	-0.590	3.01E-18	gene1620	Type III restriction endonuclease subunit R	WP_012420558.1
AMUC_RS01955	-0.593	2.32E-10	gene389	ATP-binding protein	WP_012419404.1
AMUC_RS09600	-0.593	6.92E-05	gene1912	Fe-S center ferredoxin	WP_012420831.1
AMUC_RS07810	-0.597	3.84E-11	gene1553	Phosphoribosylformimino-5-aminoimidazole carboxamide ribotide isomerase	WP_012420494.1
AMUC_RS10950	-0.598	1.14E-19	gene2183	Glutamate dehydrogenase	WP_012421075.1
AMUC_RS06385	-0.599	1.46E-14	gene1271	Hypothetical protein	WP_031930834.1
AMUC_RS08060	-0.602	1.2E-08	gene1603	DNA-directed RNA polymerase subunit alpha	WP_012420541.1
AMUC_RS10500	-0.604	7.83E-11	gene2094	Thioredoxin	WP_012420994.1
AMUC_RS01690	-0.604	1.05E-06	gene335	50S ribosomal protein L16	WP_012419353.1
AMUC_RS07515	-0.605	0.04402	gene1493	Molecular chaperone GroES	WP_012420445.1
AMUC_RS11080	-0.614	3.24E-11	gene2210	Hypothetical protein	WP_012421098.1
AMUC_RS04460	-0.614	1.47E-13	gene889	DNA-binding response regulator	WP_012419876.1

AMUC_RS01390	-0.615	1.5E-07	gene275	GNAT family acetyltransferase	WP_012419307.1
AMUC_RS01745	-0.620	0.010316	gene346	30S ribosomal protein S7	WP_012419364.1
AMUC_RS11245	-0.621	2.42E-16	gene2243	Malate dehydrogenase	WP_012421129.1
AMUC_RS01170	-0.622	1.77E-18	gene231	Glucose-1-phosphate thymidylyltransferase	WP_012419267.1
AMUC_RS01435	-0.623	1.77E-35	gene284	4-hydroxy-tetrahydrodipicolinate reductase	WP_012419315.1
AMUC_RS09465	-0.623	2.25E-31	gene1885	Glycine--tRNA ligase	WP_012420806.1
AMUC_RS05015	-0.630	1.05E-06	gene1001	50S ribosomal protein L18	WP_012419976.1
AMUC_RS09395	-0.632	5.24E-16	gene1871	ABC transporter ATP-binding protein	WP_012420792.1
AMUC_RS05010	-0.634	0.002924	gene1000	50S ribosomal protein L6	WP_012419975.1
AMUC_RS10055	-0.635	9.2E-22	gene2005	MBL fold metallo-hydrolase	WP_012420911.1
AMUC_RS03785	-0.638	4.04E-12	gene754	Beta-glucanase	WP_012419749.1
AMUC_RS02380	-0.640	5.42E-19	gene474	CinA-like protein	WP_012419483.1
AMUC_RS05005	-0.643	4.22E-16	gene999	30S ribosomal protein S8	WP_012419974.1
AMUC_RS06540	-0.644	6.88E-15	gene1301	Hypothetical protein	WP_042447975.1
AMUC_RS09155	-0.644	1.09E-09	gene1823	Succinyl-CoA ligase subunit beta	WP_012420746.1
AMUC_RS06205	-0.645	3.7E-11	gene1235	Amino acid lyase	WP_012420202.1
AMUC_RS00220	-0.654	1.37E-07	gene43	N-acetyltransferase GCN5	WP_012419101.1
AMUC_RS01735	-0.655	2.19E-08	gene344	30S ribosomal protein S10	WP_012419362.1
AMUC_RS01615	-0.655	3.21E-18	gene320	Hypothetical protein	WP_012419341.1
AMUC_RS03920	-0.658	2.58E-30	gene781	GTP-binding protein	WP_012419774.1
AMUC_RS06690	-0.659	3.27E-15	gene1330	GDP-mannose 4%2C6-dehydratase	WP_012420289.1
AMUC_RS07650	-0.661	6.98E-17	gene1521	50S ribosomal protein L31	WP_035196558.1
AMUC_RS10835	-0.683	1.69E-21	gene2160	Dihydrofolate reductase	WP_012421055.1
AMUC_RS09135	-0.692	2.57E-17	gene1819	Recombinase RecQ	WP_012420743.1
AMUC_RS05795	-0.701	1.23E-12	gene1153	23S rRNA (guanosine(2251)-2'-O)-methyltransferase RlmB	WP_012420121.1
AMUC_RS11880	-0.704	1.29E-10	gene875	Hypothetical protein	WP_052294442.1
AMUC_RS02675	-0.716	3.12E-25	gene533	Fe-S cluster assembly ATPase SufC	WP_035196050.1
AMUC_RS05195	-0.716	2.85E-13	gene1037	Hypothetical protein	WP_051729423.1
AMUC_RS02010	-0.735	1.06E-48	gene400	Hypothetical protein	WP_042447573.1
AMUC_RS02670	-0.739	4.76E-12	gene532	Transcriptional repressor	WP_012419539.1
AMUC_RS08815	-0.744	4.18E-07	gene1755	Secretion protein	WP_012420685.1
AMUC_RS01750	-0.746	5.21E-12	gene347	30S ribosomal protein S12	WP_012419365.1
AMUC_RS04995	-0.769	4.3E-22	gene997	50S ribosomal protein L24	WP_012419972.1
AMUC_RS00740	-0.779	7.93E-22	gene147	Transposase	WP_012419186.1
AMUC_RS10155	-0.779	6.39E-15	gene2026	Nitrogen-fixing protein NifU	WP_012420930.1
AMUC_RS04320	-0.784	0.017267	gene861	Cupin	WP_012419849.1
AMUC_RS01360	-0.793	5.73E-22	gene269	Trna (guanine(37)-N(1))-methyltransferase	WP_042448603.1
AMUC_RS05000	-0.795	8.76E-10	gene998	50S ribosomal protein L5	WP_012419973.1
AMUC_RS06140	-0.804	3.1E-07	gene1222	Potassium-transporting ATPase subunit KdpA	WP_022196803.1
AMUC_RS05250	-0.807	7.34E-19	gene1048	Hypothetical protein	WP_012420024.1

AMUC_RS06035	-0.812	5.83E-13	gene1201	Hypothetical protein	WP_012420169.1
AMUC_RS03790	-0.814	2.43E-17	gene755	Beta-glucanase	WP_012419750.1
AMUC_RS02580	-0.834	1.11E-25	gene514	N-acetyltransferase GCN5	WP_012419522.1
AMUC_RS01595	-0.836	6.37E-15	gene316	Ribonuclease HIII	WP_042448620.1
AMUC_RS09680	-0.858	3.1E-11	gene1928	Hypothetical protein	WP_042448307.1
AMUC_RS05935	-0.861	1.41E-21	gene1181	ATP phosphoribosyltransferase	WP_012420148.1
AMUC_RS08820	-0.894	2.28E-38	gene1756	Phosphatidylserine decarboxylase	WP_012420686.1
AMUC_RS02895	-0.896	2.05E-24	gene577	Glutamate 5-kinase	WP_012419582.1
AMUC_RS06355	-0.926	3.22E-57	gene1265	Alcohol dehydrogenase	WP_012420230.1
AMUC_RS11295	-0.932	2.99E-51	gene2253	Threonine dehydrogenase	WP_012421139.1
AMUC_RS09030	-0.941	3.91E-22	gene1798	Hypothetical protein	WP_042448236.1
AMUC_RS08450	-0.948	4.78E-22	gene1682	Nucleoside-diphosphate kinase	WP_012420615.1
AMUC_RS06345	-0.957	7.49E-26	gene1263	50S ribosomal protein L28	WP_012420229.1
AMUC_RS02660	-1.006	2.49E-23	gene530	50S ribosomal protein L21	WP_012419537.1
AMUC_RS06145	-1.017	1.45E-06	gene1223	Potassium-transporting ATPase subunit B	WP_012420192.1
AMUC_RS09380	-1.039	3E-79	gene1868	Phosphocarrier protein Hpr	WP_012420789.1
AMUC_RS01260	-1.043	2.12E-25	gene249	Hypothetical protein	WP_042447516.1
AMUC_RS01425	-1.051	1.07E-31	gene282	3-methyl-2-oxobutanoate hydroxymethyltransferase	WP_012419313.1
AMUC_RS09840	-1.084	2.88E-50	gene1961	ECF subfamily RNA polymerase sigma-24 subunit	WP_012420873.1
AMUC_RS06150	-1.095	3.31E-08	gene1224	Potassium transporter KtrB	WP_012420193.1
AMUC_RS01355	-1.225	5.07E-68	gene268	Hypothetical protein	
AMUC_RS07395	-1.303	3.58E-27	gene1470	Short-chain dehydrogenase/reductase SDR	WP_012420428.1
AMUC_RS00015	-1.317	5.85E-44	gene2	NUDIX hydrolase	WP_012419063.1
AMUC_RS00360	-1.343	1.18E-10	gene72	Membrane protein	WP_042448508.1

Log2Fold Change = ox-bile / control condition (n = 3; the experiment was performed in triplicate).

Padj was calculated using the procedure of Benjamini and Hochberg. The upregulated and downregulated genes under ox-bile condition (padj < 0.05) were listed.
